# The Impact of Nicotine along with Oral Contraceptive Exposure on Brain Fatty Acid Metabolism in Female Rats

**DOI:** 10.3390/ijms232416075

**Published:** 2022-12-16

**Authors:** Shahil H. Patel, Alba Timón-Gómez, Hari Pradhyumnan, Berk Mankaliye, Kunjan R. Dave, Miguel A. Perez-Pinzon, Ami P. Raval

**Affiliations:** 1 Peritz Scheinberg Cerebral Vascular Disease Research Laboratories, Leonard M. Miller School of Medicine, University of Miami, Miami, FL 33136, USA; 2 Department of Neurology, Leonard M. Miller School of Medicine, University of Miami, Miami, FL 33136, USA; 3 Neuroscience Program, Leonard M. Miller School of Medicine, University of Miami, Miami, FL 33136, USA; 4 Bruce W. Carter Department of Veterans Affairs Medical Center, Miami, FL 33136, USA

**Keywords:** nicotine, oral contraceptive, β-oxidation, carnitine palmitoyltransferase enzymes 1 and 2, metabolomics, phospholipids, phosphatidylcholine, stroke, palmitoylation

## Abstract

Smoking-derived nicotine (N) and oral contraceptive (OC) synergistically exacerbate ischemic brain damage in females, and the underlying mechanisms remain elusive. In a previous study, we showed that N + OC exposure altered brain glucose metabolism in females. Since lipid metabolism complements glycolysis, the current study aims to examine the metabolic fingerprint of fatty acids in the brain of female rats exposed to N+/−OC. Adolescent and adult Sprague–Dawley female rats were randomly (n = 8 per group) exposed to either saline or N (4.5 mg/kg) +/−OC (combined OC or placebo delivered via oral gavage) for 16–21 days. Following exposure, brain tissue was harvested for unbiased metabolomic analysis (performed by Metabolon Inc., Morrisville, NC, USA) and the metabolomic profile changes were complemented with Western blot analysis of key enzymes in the lipid pathway. Metabolomic data showed significant accumulation of fatty acids and phosphatidylcholine (PC) metabolites in the brain. Adolescent, more so than adult females, exposed to N + OC showed significant increases in carnitine-conjugated fatty acid metabolites compared to saline control animals. These changes in fatty acyl carnitines were accompanied by an increase in a subset of free fatty acids, suggesting elevated fatty acid β-oxidation in the mitochondria to meet energy demand. In support, β-hydroxybutyrate was significantly lower in N + OC exposure groups in adolescent animals, implying a complete shunting of acetyl CoA for energy production via the TCA cycle. The reported changes in fatty acids and PC metabolism due to N + OC could inhibit post-translational palmitoylation of membrane proteins and synaptic vesicle formation, respectively, thus exacerbating ischemic brain damage in female rats.

## 1. Introduction

Stroke and the resulting consequence of cerebral ischemic pathology are sexually dimorphic, with females showing more severe stroke outcomes than males [1,2,3,4]. One of the female-specific factors that has been shown to increase the risk and severity of stroke is use of either oral contraceptive (OC) or hormone replacement therapy (HRT) during reproductive age or menopause, respectively [1]. Ample literature has shown that long-term exposure to HRT or OC affects blood clotting, lipid and lipoprotein metabolism, and endothelial function [5]. In contrast, a recent study shows that both OC use and HRT were associated with an increased risk of stroke, especially during the first year of use, possibly due to immediate changes in hemostatic balance [6]. It is also documented that OC exposure makes females more susceptible to stress hormone effects on episodic memory, fear conditioning, and cognitive emotion regulation [7,8,9,10]. These detrimental effects of OC in association with comorbidities such as polycystic ovary syndrome, exposures to endocrine disrupting chemicals, and especially smoking-derived nicotine (N) exposure exacerbate both the risk and severity of stroke [5]. Over the past decade, battery-powered nicotine-delivery devices known as electronic cigarettes (EC) have gained popularity amongst young girls and women who are smokers actively trying to give up smoking [11]. Since EC deliver nicotine, identifying the effects of nicotine on cerebral fat metabolism can also help delineate its effects on the brain. Understanding the effects of EC on the brain is important because, given the short life of EC in the market, our understanding is limited.

Nicotine’s effect on the brain differs between the sexes, predominantly because nicotine is metabolized faster in females than it is in males [12]. Additionally, nicotine retards the conversion of testosterone into estradiol as an inhibitor of the enzyme aromatase, leading to low levels of circulating 17β-estradiol [13,14]. Low levels of circulating estradiol have been implicated in increased cerebral ischemic brain damage and N alone, or in combination with OC, has been shown to decrease availability of membrane-bound and mitochondrial estrogen receptor subtype beta (ER-β) in the female rat brain [14,15,16]. Since mitochondrial ER-β regulates the activity of cytochrome c oxidase, the terminal enzyme of oxidative phosphorylation, N + OC-induced loss of mitochondrial ER-β reduced the activity of cytochrome c oxidase leading to mitochondrial dysfunction [16]. In a separate study employing an unbiased global metabolomic approach, we further demonstrated that N + OC exposure altered cerebral glycolysis; in particular, N + OC exposure significantly decreased glucose, glucose 6-phosphate, and fructose-6-phosphate levels, while significantly increasing the level of pyruvate [17]. These changes in glycolysis were preeminent in the brains of adolescent versus adult rats, suggesting the age-dependent effects of N + OC combination and their impact on stroke outcomes [17]. As the observed alterations in the glycolytic pathway can influence lipid metabolism, the goal of the current study is to investigate the synergistic effects of N + OC on fatty acid and phospholipid metabolism in the brain of adolescent and adult female rats using an unbiased global metabolomic approach.

## 2. Methods

Animals: The experimental procedures were all performed in accordance with the Guide for the Care and Use of Laboratory Animals published by the National Institutes of Health, as well as University of Miami Animal Care and Use Committee approved protocols. Additionally, the results are communicated as per the Animal Research: Reporting of In Vivo Experiments guidelines. Adolescent (6-week-old) and adult (14-week-old) female Sprague–Dawley rats were used, and rats showing three normal estrous cycles (approximately 4 days) in succession were included for experiments. The experimental groups consisted of n = 8 rats in two age groups that were randomly allocated. A total of 64 rats were used for the current experiments and no rats were excluded from the study.

Nicotine exposure: Nicotine was delivered using an osmotic pump (Alzet Corp., Palo Alto, CA) in the form of nicotine hydrogen tartrate delivered in a continuous fixed rate of 4.5 mg/kg/day, which is equivalent to 1.5 mg/kg/day of free base nicotine [14]. Control-group pumps administered only saline (S). Both groups received exposure for 16–21 days.

Oral contraceptive treatment: Combined OCs are popular and contain ethinyl estradiol plus progesterone. Therefore, the current study used combined OC pills [18]. OC treatment was administered for approximately 4 estrous cycles for a total of 16–21 days, as described in our previous publication [14]. Both OC and placebo tablet treatment were administered orally for three consecutive days determined by the 4-day estrous cycle of rats in order to resemble OC usage in women [14].

Global Metabolomic Analysis: A global metabolomic profile was performed on the cortical tissue obtained from rats in S, N, OC, and N + OC exposure groups [17]. The unbiased global metabolomic procedures and data analysis were performed by Metabolon Inc. (Morrisville, NC, USA), and the details remain the same as published previously [19]. Briefly, the global metabolomic profile of the collected tissue was determined using a Waters ACQUITY ultraperformance liquid chromatography (UPLC) and a Thermo Scientific Q-Exactive high-resolution/accurate mass spectrometer interfaced with a heated electrospray ionization (HESI-II) source and Orbitrap mass analyzer. Metabolon Inc.’s library of purified standards of known retention time/index (RI), mass-to-charge ratio (m/z), and chromatography data were used to determine the identity of the compounds found in the samples by comparison. Proprietary software and procedures were employed by Metabolon Inc. to ensure high-quality data and accurate compound identification. The quantification of metabolites was calculated using the area under the peak normalized by sample protein content. Metabolon Inc. used ArrayStudio, Program R, and JMP for bioinformatic analyses, significance testing, and classification analyses.

**Figure 1:**
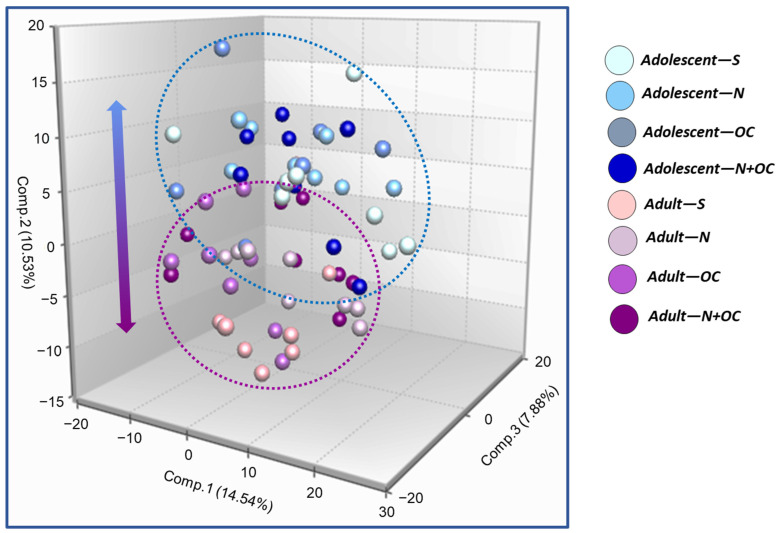
PCA analysis shows clustering of cortical samples from female rats exposed to N, OC, or N + OC cluster by age (adolescent vs. adult), with modest separation of samples by experimental treatment (saline vs. N, OC, or N + OC) in the adolescent group, which is suggestive of age-dependent metabolic changes.

**Figure 2:**
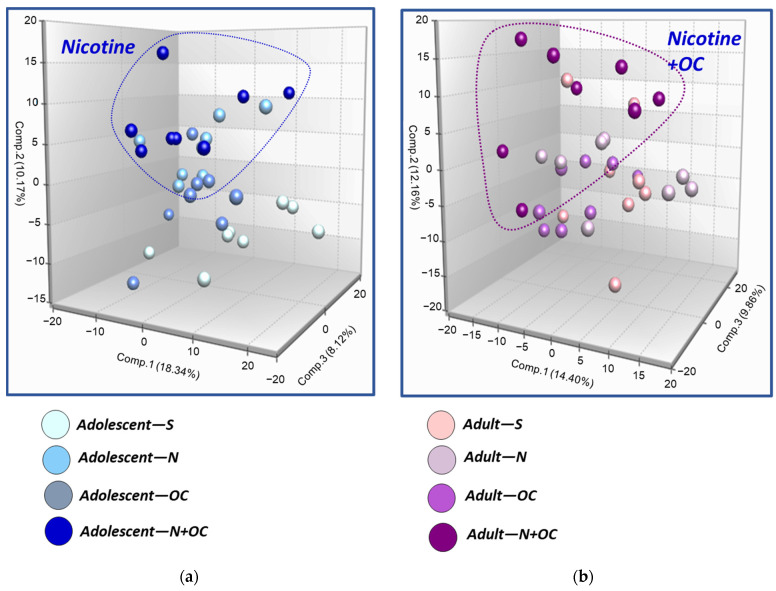
PCA analysis after further separation by age group (adolescent vs. adult) shows that (**a**) adolescent animal samples clustered by experimental treatment as N- or N + OC-exposed groups clustered apart from the saline-treated group, indicating distinction in cortical metabolic profiles with N and N + OC treatment compared to saline treatment. Saline and OC experimental groups showed minor overlap in clustering. (**b**) Adult animal samples showed less segregation than adolescent animals based on experimental treatment (saline vs. N, OC, or N + OC).

Principal Component Analysis (PCA): PCA reduces the dimensionality of the dataset by creating a linear combination of every metabolite to view similarities and differences between experimental groups. The first principal component is computed by determining the coefficients of the metabolites that maximize the variance of the linear combination. The second component finds the coefficients that maximize the variance with the condition that the second component is orthogonal to the first. The third component is orthogonal to the first two components, and so on. The total variance is defined as the sum of the variances of the predicted values of each component (the variance is the square of the standard deviation), and for each component the proportion of the total variance is computed. These data are provided in Figure 1 and Figure 2.

Western blotting: As described in our previous publications, protein content of the cortical tissue was determined after the tissue was homogenized, and the proteins were separated by 12% stain-free sodium dodecyl sulfate–polyacrylamide gel electrophoresis (SDS–PAGE) [17]. These separated proteins were then transferred to a polyvinylidene difluoride (PVDF) membrane so that they could be incubated with the following primary antibodies: anti-choline kinase α (CKα; 1:15,000; catalog # ab88053; Abcam), anti-phosphate cytidylyltransferase 1 B (PCYT1B, CCTβ; 1:1000; catalog # NBP2-19734; Novus Biologicals), anti-p44/42 mitogen-activated protein kinase (MAPK; 1:1500; catalog # 9102S; Cell Signaling), anti-carnitine palmitoyltransferase 1 A (CPT1A; 1:1000; catalog # ABS65; Millipore/Sigma), anti-carnitine palmitoyltransferase 2 (CPT2; 1:1000; catalog # ABS85; Millipore/Sigma), anti-acyl-CoA synthetase (ACSL; 1:1000; catalog # 4047; Cell Signaling), anti-malonyl-CoA decarboxylase (MLYCD; 1:1000; catalog # 15265-1-AP; Proteintech), anti-ATP citrate lyase (ACL; 1:1000; catalog # 4332; Cell Signaling), and anti-fatty acid synthase (FASN; 1:1000; catalog # 3180; Cell Signaling). All Western blot data were normalized to β-actin loading control (monoclonal; 1:1000; catalog # A2228; Millipore/Sigma), and all imaging was performed using the ChemiDoc MP Imaging System (BioRad). A digital densitometric analysis was then performed on the produced immunoblots using FIJI software [17].

Statistical analysis: Western blot was performed using 4–6 samples and immunoblot images were analyzed by an investigator blinded to the conditions of the experiment. One-way ANOVA was used to determine mean differences between multiple groups, and the data are presented as mean ± SD. For the metabolomic data, *p*- and q-values were calculated using a three-way ANOVA analysis. The significant data are represented in box plots. In the box plot, extreme data points are represented as circles; maximum and minimum distribution are represented with error bars; the median value is represented with a horizontal line; upper and lower quartiles are represented by the upper and lower borders of the box, and the mean value is denoted with a “+” sign inside the box.

## 3. Results

N + OC treatment segregated samples by both treatment and age: As shown in Figure 1, PCA analysis was successful in segregating the cortical samples by age (adolescent vs. adult), with modest separation of samples by nicotine treatment (saline vs. N, OC, or N + OC) in the adolescent group, perhaps suggesting that differences in cortical metabolic profile is driven primarily by age. Additional PCAs with further stratification of samples by age (adolescent and adult) demonstrated clear segregation of N and N + OC samples from saline samples, with minor overlap between OC and saline samples in adolescent rodents, possibly reflecting profound differences in cortical metabolic profiles with N and N + OC treatment (in relation to saline treatment), as shown in Figure 2a. In comparison, adult samples showed limited segregation of samples based on treatment (saline vs. N, OC, or N + OC), probably indicating subtle differences in cortical metabolic profiles in adults after treatment (Figure 2b). Limited segregation of samples between treatment groups can arise from collection effects, or greater variation in response to different treatments within a group or age-related differences in response.

**Figure 3:**
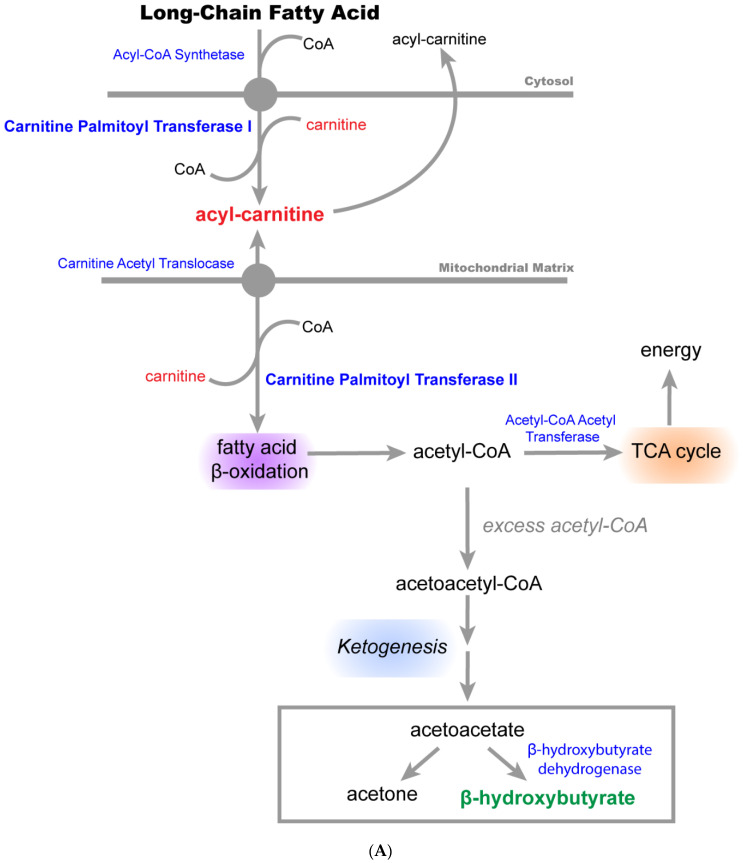

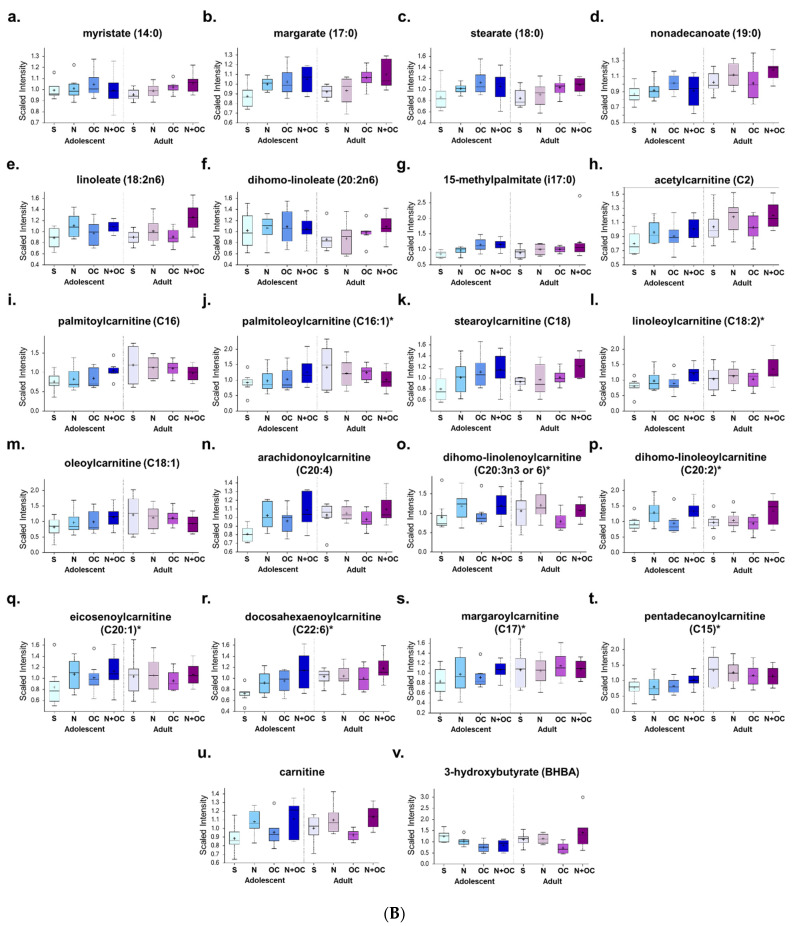
N + OC alters lipid metabolism metabolites, particularly fatty acid oxidation metabolites, in the cortex of adolescent and adult female rats. (**A**) Depicts the fatty acid oxidation pathway that takes place in the mitochondria. The red font indicates a significant increase in the metabolite, and green font indicates a significant decrease. (**B**) Box plots showing that N + OC alters lipid metabolism metabolites, particularly fatty acid oxidation metabolites in the cortex of adolescent and adult female rats. (**a**–**v**) These box plots show significant alterations in myristate (14:0), margarate (17:0), stearate (18:0), nonadecanoate (19:0), linoleate (18:2n6), dihomo-linoleate (20:2n6), 15-methylpalmitate (i17:0), acetylcarnitine (C2), palmitoylcarnitine (C16), palmitoleoylcarnitine (C16:1)*, stearoylcarnitine (C18), linoleoylcarnitine (C18:2)*, oleoylcarnitine (C18:1), arachidonoylcarnitine (C20:4), dihomo-linolenoylcarnitine (C20:3n3 or 6)*, dihomo-linoleoylcarnitine (C20:2)*, eicosenoylcarnitine (C20:1)*, docosahexaenoylcarnitine (C22:6)*, margaroylcarnitine (C17)*, pentadecanoylcarnitine (C15)*, carnitine, and BHBA fold change in the cortex of saline-, N-, OC-, and N  +  OC-exposed rats. “Biochemical name*” indicates a compound that has not been confirmed based on a standard, but Metabolon is confident in its identity.

N + OC alters fatty acid oxidation in the brain: One of the prominent changes in the dataset was elevated levels of carnitine-conjugated fatty acid metabolites (Figure 3 and Supplementary Tables S1, S3 and S4) such as myristate (14:0), margarate (17:0), stearate (18:0), nonadecanoate (19:0), linoleate (18:2n6), dihomo-linoleate (20:2n6), 15-methylpalmitate (i17:0), acetylcarnitine (C2), palmitoylcarnitine (C16), palmitoleoylcarnitine (C16:1), stearoylcarnitine (C18), linoleoylcarnitine (C18:2), oleoylcarnitine (C18:1), arachidonoylcarnitine (C20:4), dihomo-linolenoylcarnitine (C20:3n3 or 6), dihomo-linoleoylcarnitine (C20:2), eicosenoylcarnitine (C20:1), docosahexaenoylcarnitine (C22:6), margaroylcarnitine (C17), pentadecanoylcarnitine (C15), and carnitine in N-, OC-, and N + OC-exposed adolescent animals when compared to the saline-exposed animals. These changes were most markedly observed with N + OC exposure, although adult animals showed limited changes in acyl carnitines with treatment. Changes in fatty acyl carnitines were accompanied by an increase in a subset of free fatty acids (FFA) in N-, OC-, and N + OC-exposed adolescent animals. Furthermore, β-hydroxybutyrate (BHBA) levels were significantly lower in OC- and N + OC-exposed adolescent animals as compared to the respective saline group. Despite increases in a subset of fatty acids in OC and N + OC adult animal groups, subsequent increases in carnitine-conjugated metabolites were not observed.

**Figure 4:**
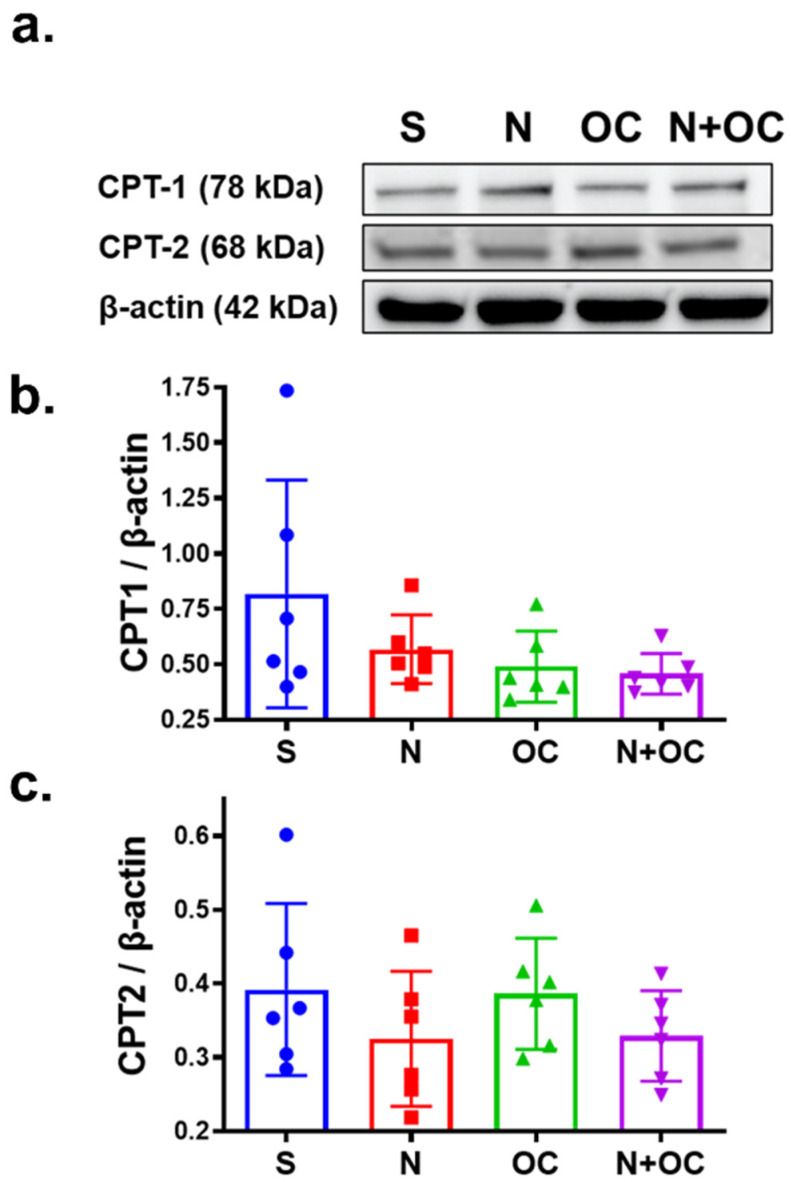
Immunoblotting of key enzymes involved in fatty acid metabolism, and in particular, β-oxidation. (**a**) Immunoblots show steady-state protein levels of carnitine palmitoyltransferase I (CPT1) and carnitine palmitoyltransferase II (CPT2) in the cortex of saline-, N-, OC-, and N  +  OC-exposed rats. Β-actin was the loading control. (**b**,**c**) Western blot analysis demonstrates lower steady-state levels of CPT1 and CPT2 in N- and N + OC-exposed rats as compared to saline-exposed rats; however, the differences are not significant.

The process of deriving energy in the form of ATP via fatty acids and their oxidation is mediated by the carnitine palmitoyltransferase (CPT) system and involves two separate enzymes located in the outer (CPT1) and inner (CPT2) mitochondrial membranes. Since we observed alterations in the carnitine-conjugated fatty acid metabolites, we analyzed the protein levels of these enzymes in the cortexes of adolescent female rats using Western blotting. Western blot analysis revealed no significant changes in either CPT1 or CPT2 protein levels amongst different experimental groups (Figure 4).

**Figure 5:**
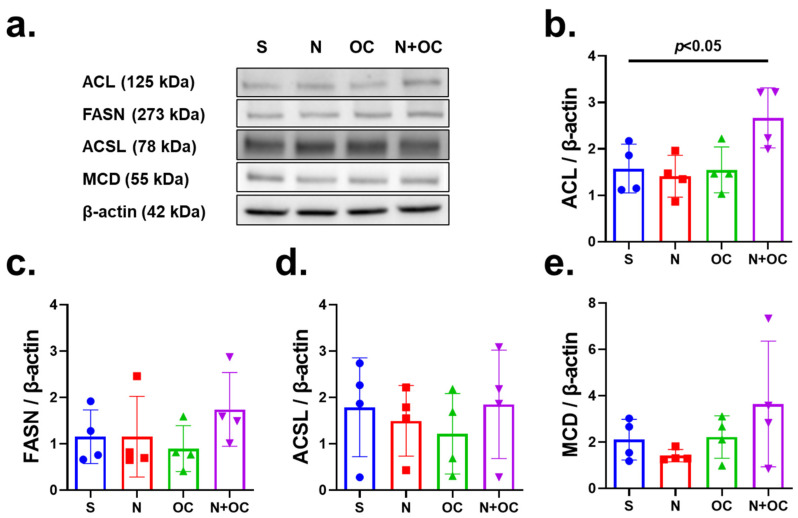
Immunoblotting of enzymes involved in fatty acid metabolism. (**a**) Immunoblots show steady-state protein levels of ATP-citrate lyase (ACL), fatty acid synthase (FASN), acyl-CoA synthetase (ACSL), and malonyl-CoA decarboxylase (MCD) in the cortex of saline-, N-, OC-, and N  +  OC-exposed rats. Β-actin was the loading control. (**b**) Western blot analysis demonstrates a significant increase (*p* < 0.05) in protein expression levels of ACL in the N + OC treatment group. (**c**–**e**) No significant changes due to experimental exposure in steady-state levels of FASN, ACSL, or MCD were found.

Since the enzyme acyl-CoA synthetase (ACSL) catalyzes the ligation reaction of CoA esters to FFAs, which is crucial for fatty acids to be channeled for use in β-oxidation [20], next we investigated the steady-state protein level of ACSL. The protein levels of ACSL did not show significant change in the treatment groups (Figure 5a,d), suggesting that fatty acids may not be shuttled to β-oxidation through this metabolic pathway. Furthermore, the protein levels of fatty acid synthase (FASN) and malonyl-CoA decarboxylase (MCD) did not change due to either N or N + OC exposure (Figure 5a,c,e). FASN is the rate-limiting enzyme that catalyzes the formation of long-chain fatty acids from acetyl-CoA and malonyl-CoA, while MCD is responsible for the degradation of malonyl-CoA [21]. As reported previously, TCA Cycle metabolites, viz.,-succinate, aconitate, and citrate, were significantly increased due to N + OC. Citrate is converted to acetyl-CoA via the enzyme ATP-citrate lyase (ACL), which would feed acetyl-CoA into the fatty acid synthesis pathways to facilitate the observed accumulation of FFA in the brains of rats exposed to N + OC. Protein expression of ACL was significantly increased in N + OC-exposed animals, suggesting that increased acetyl-CoA supply for FFA synthesis could be the cause of the observed accumulation (Figure 5a,b).

**Figure 6:**
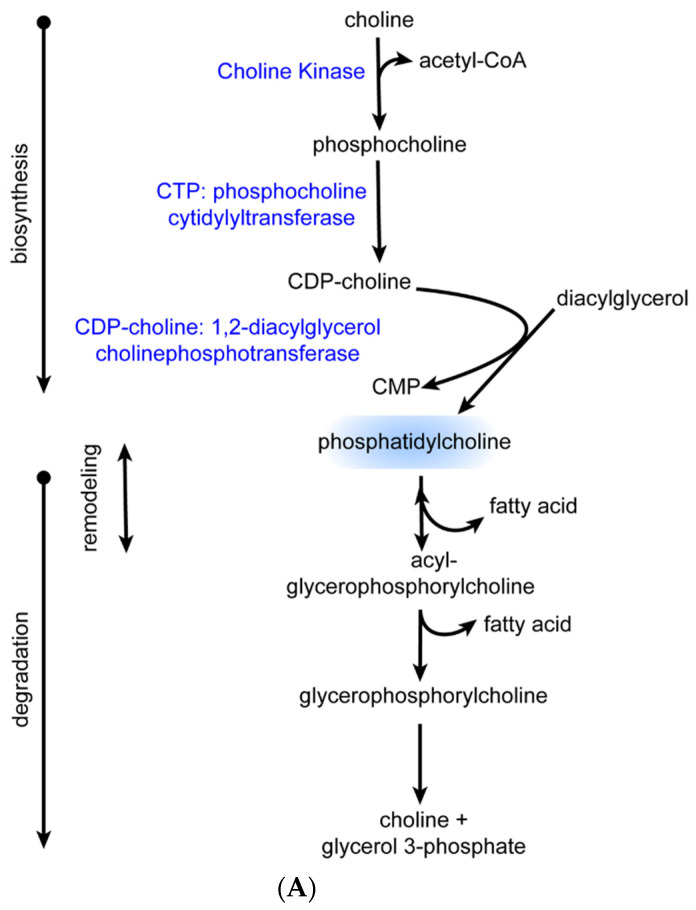

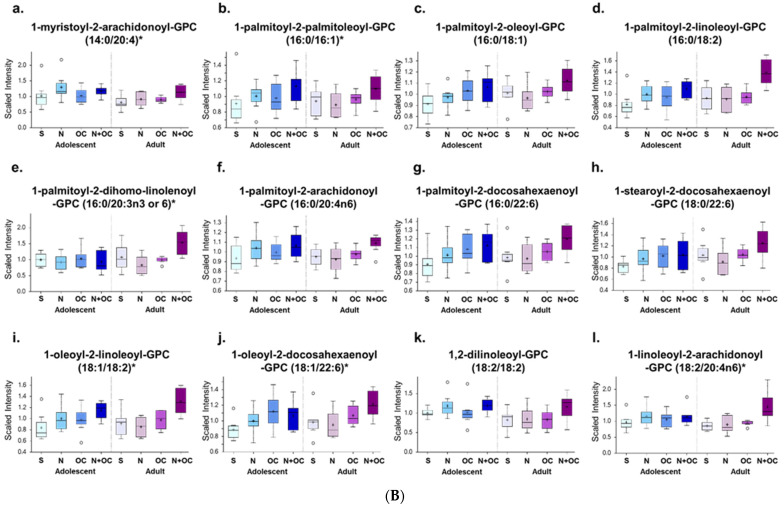
N + OC alters PC metabolites in the cortex of adolescent female rats. (**A**) The biosynthesis (Kennedy pathway), remodeling, and degradation that takes place in relation to PC metabolism. (**B**) Box plots showing that N + OC alters PC metabolites. (**a**–**l**) Box plots show significant alterations in 1-myristoyl-2-arachidonoyl-GPC (14:0/20:4)*, 1-palmitoyl-2-palmitoleoyl-GPC (16:0/16:1)*, 1-palmitoyl-2-oleoyl-GPC (16:0/18:1), 1-palmitoyl-2-linoleoyl-GPC (16:0/18:2), 1-palmitoyl-2-dihomo-linolenoyl-GPC (16:0/20:3n3 or 6)*, 1-palmitoyl-2-arachidonoyl-GPC (16:0/20:4n6), 1-palmitoyl-2-docosahexaenoyl-GPC (16:0/22:6), 1-stearoyl-2-docosahexaenoyl-GPC (18:0/22:6), 1-oleoyl-2-linoleoyl-GPC (18:1/18:2)*, 1-oleoyl-2-docosahexaenoyl-GPC (18:1/22:6)*, 1,2-dilinoleoyl-GPC (18:2/18:2), and 1-linoleoyl-2-arachidonoyl-GPC (18:2/20:4n6)* fold change in the cortex of saline-, N-, OC-, and N  +  OC-exposed rats. “Biochemical name*” indicates a compound that has not been confirmed based on a standard, but Metabolon is confident in its identity.

**Figure 7:**
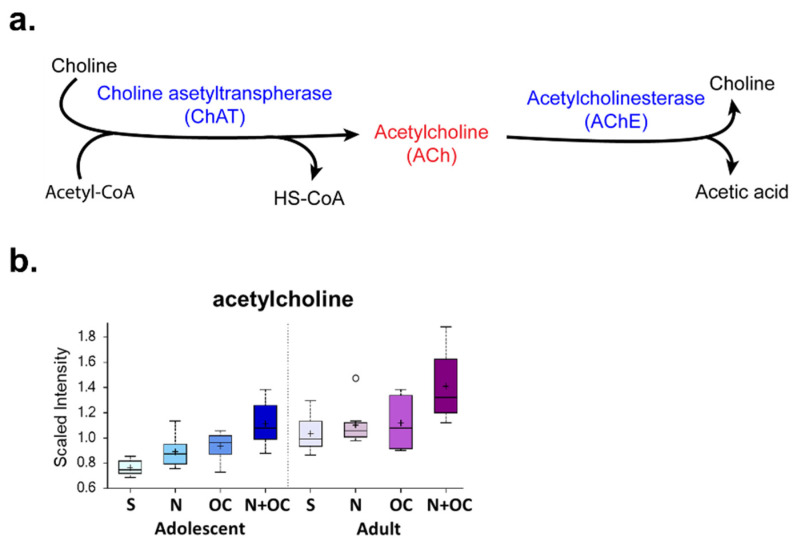
N + OC increases acetylcholine levels in the cortex of adolescent female rats. (**a**) Biosynthesis and degradation of the neurotransmitter acetylcholine. (**b**) The table shows fold change of acetylcholine; the red colored boxes represent a significant increase (*p* ≤ 0.05) in that metabolite.

N + OC induced phosphatidylcholine (PC) accumulation: Since PC is the most abundant phospholipid in cell membranes and also acts as a reservoir for choline, thus being implicated in acetylcholine synthesis, exploring any effect on PC due to N + OC is essential [22,23]. We observed elevated PC levels in N, OC, and N + OC groups as compared to the saline group (Figure 6 and Supplementary Table S5a,b). Alterations were observed in levels of 1-myristoyl-2-arachidonoyl-GPC (14:0/20:4), 1-palmitoyl-2-palmitoleoyl-GPC (16:0/16:1), 1-palmitoyl-2-oleoyl-GPC (16:0/18:1), 1-palmitoyl-2-linoleoyl-GPC (16:0/18:2), 1-palmitoyl-2-dihomo-linolenoyl-GPC (16:0/20:3n3 or 6), 1-palmitoyl-2-arachidonoyl-GPC (16:0/20:4n6), 1-palmitoyl-2-docosahexaenoyl-GPC (16:0/22:6), 1-stearoyl-2-docosahexaenoyl-GPC (18:0/22:6), 1-oleoyl-2-linoleoyl-GPC (18:1/18:2), 1-oleoyl-2-docosahexaenoyl-GPC (18:1/22:6), 1,2-dilinoleoyl-GPC (18:2/18:2), and 1-linoleoyl-2-arachidonoyl-GPC (18:2/20:4n6). The observed PC accumulation was more pronounced in the N + OC-exposed group, and the accumulation did not show much age-dependent variation as both adolescent and adult brains showed elevated PC levels as compared to respective saline groups. Most importantly, acetylcholine levels were uniformly elevated with N, OC, and N + OC exposure in adolescent rats versus saline exposure (Figure 7), which is confirmatory of the direct effects of N+/−OC on neurotransmission.

**Figure 8:**
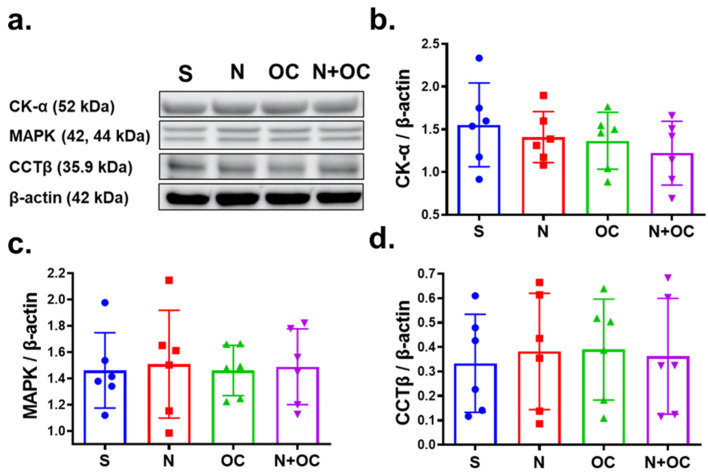
Immunoblotting of key enzymes involved in PC metabolism. (**a**) Immunoblots show steady-state protein levels of choline kinase α (CKα), mitogen-activated protein kinase (MAPK), and phosphocholine cytidylyltransferase β (CCTβ) in the cortex of saline-, N-, OC-, and N  +  OC-exposed rats. β-actin was the loading control. (**b**–**d**) Western blot analysis demonstrates no significant changes in steady-state protein levels for CKα, MAPK, and CCTβ.

In the brain, phospholipids are generated from acylated lipids, and membrane-derived phospholipids control synaptic neurotransmission and plasticity [24]. PC is produced via the CDP–choline–Kennedy pathway and is synthesized from choline (Figure 6). The first of three reactions of this pathway is the synthesis of phosphocholine from choline, which is carried out by the cytosolic phosphotransferase Choline Kinase (CKα). The following enzyme in the CDP–choline–Kennedy pathway is CTP:phosphocholine cytidylyltransferase (CCT), which is responsible for next converting phosphocholine to CDP-choline. This conversion catalyzed by CCT is the rate-limiting step in the CDP–choline–Kennedy pathway. CCT has two isoforms, CCTα and CCTβ. CCTβ is the most abundant isoform in brain tissue and the transcription of the genes that encode both of the isoforms of CCT is regulated by the MAP extracellular signal-regulated kinases 1/2 (ERK1/2) pathway (MAPK) [25,26]. The third reaction of the CDP–choline–Kennedy pathway is the condensation reaction between CDP–choline and diacylglycerol (DAG) to form PC, and it is catalyzed by CDP–choline:1,2-diacylglycerol cholinephosphotransferase. Since the PC synthesis from choline occurs in three major reactions carried out by three distinct enzymes (Figure 6), CKα, CCTβ, and MAPK, we monitored protein levels of these enzymes. Western blot analysis revealed no changes in the protein levels of any of these enzymes with experimental exposures under investigation (Figure 8).

## 4. Discussion

Approximately 11.6 million American women are users of OC of which one-fourth also smoke cigarettes, and it has been shown that incidence and severity of stroke increases in those who combine OC with cigarette smoking [27,28,29]. Therefore, understanding the mechanism responsible for increased severity of ischemic brain damage because of these comorbidities is crucial and in accordance with the STAIR consortium recommendations [30]. Studies have shown that even a short exposure of 2–3 weeks of N + OC causes significant deficits in synaptic function, cerebral blood flow, and energy metabolism, thus exacerbating ischemic brain damage [17,31,32]. Studies also demonstrated that N alone, or in combination with OC, altered key metabolites in the glycolytic pathway [17]. For example, N + OC exposure significantly decreased glucose, glucose-6-phosphate, and fructose-6-phosphate levels, whereas data showed significant increase in pyruvate levels. The metabolomic findings showing defects in glycolysis after N + OC exposure were also corroborated by decreased availability and enzyme activity of hexokinase, the rate-limiting enzyme of the glycolytic pathway [17]. Furthermore, observed defects in glycolysis after N + OC were also reflected in increased levels of TCA cycle metabolites, viz.,- aconitate, succinate, and citrate. The citrate is converted to FFA via acetyl-CoA. Here, we present data showing N + OC exposure causes accumulation of FFA, PC, and acetylcholine in female rat brains (Figure 7). Since nicotine is a neuronal nicotinic acetylcholine receptor (nAChR) agonist and is also known to decrease acetylcholinesterase activity, the metabolomic data furthermore confirm this well-established fact by showing that N alone or in combination with OC significantly increases the levels of acetylcholine in the brain of female rats [33].

**Figure 9:**
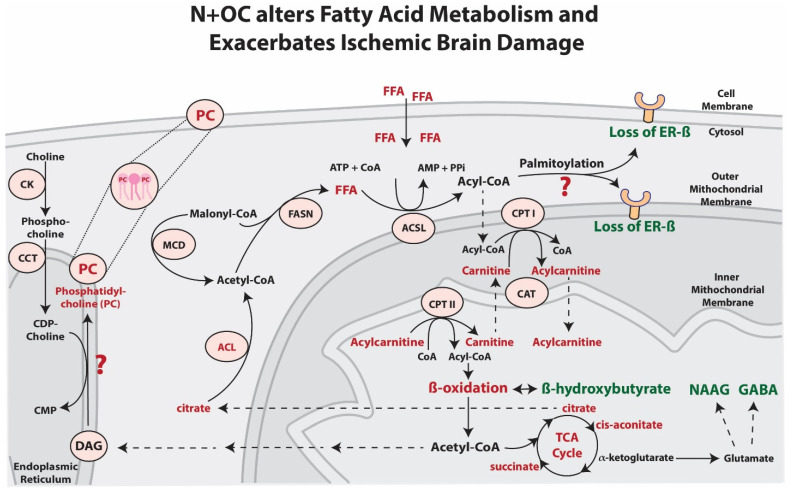
Putative effects of N + OC exposure on lipid metabolism leading to severe ischemic stroke outcomes. Our findings indicate that N + OC elicits an increase in free fatty acids (FFA). Acyl-CoA synthetase (ACSL) is responsible for converting FFA into acyl-CoAs at the outer mitochondrial membrane. Acyl-CoAs that pass into the mitochondria donate their acyl groups to carnitine to form acylcarnitine in a reaction facilitated by carnitine palmitoyltransferase I (CPT I). This allows carnitine in the form of acylcarnitine to be shuttled through the inner mitochondrial membrane via carnitine acylcarnitine translocase (CAT). These acylcarnitines are then converted back to carnitine by carnitine palmitoyltransferase II (CPT II). Acyl-CoA is regenerated in this mechanism inside the mitochondria where it can then be used for energy production via β-oxidation and the TCA cycle. N + OC exposure decreased levels of the ketone body β-hydroxybutyrate, suggesting that exposure to N + OC potentially initiated an energetic shift to fat metabolism. The TCA metabolite α-ketoglutarate is especially relevant as it is a primary source of synthesis for the neurotransmitter glutamate [58]. Glutamate is the precursor of the neurotransmitters N-Acetylaspartylglutamic acid (NAAG) as well as γ-Aminobutyric acid (GABA). The TCA metabolite citrate is transported outside of the mitochondria and the enzyme ATP-citrate lyase (ACL) subsequently breaks it down into acetyl-CoA and oxaloacetate. Another source of cytosolic acetyl-CoA is from the degradation of malonyl-CoA into acetyl-CoA and carbon dioxide by the enzyme malonyl-CoA decarboxylase (MCD). The enzyme fatty acid synthase (FASN) takes malonyl-CoA and acetyl-CoA and catalyzes the formation of long-chain free fatty acids. Changes in FFA levels affecting Acyl-CoA can subsequently disrupt processes dependent on these molecules such as palmitoylation, which utilizes palmitoyl-CoA as its main acyl-CoA substrate. Estrogen receptor subtype beta (ER-β), a receptor previously implicated in neuroprotection after ischemic damage, requires palmitoylation to function [14]. Altered palmitoylation after exposure to N + OC may exacerbate ischemic brain damage. In the brain, alterations to phosphatidylcholine (PC) homeostasis due to N + OC may be of consequence as PC is the most abundant phospholipid found in cell membranes. Our study found no changes in phosphocholine cytidylyltransferase β (CCTβ) or choline kinase α (CKα) levels in the PC synthesis pathway. Possible changes in the reaction converting to PC caused by N + OC could be the root cause of the observed increase in PC levels. Metabolites and processes depicted in green font indicate a statistically significant decrease (*p* ≤ 0.05) in levels. Metabolites and processes depicted in red font indicate a statistically significant increase (*p* ≤ 0.05) in levels. ?: how the effects of N + OC on palmitoylation and PC synthesis in the context of stroke are not known.

The metabolomic data showed a significant decrease in BHBA concentrations in the cortical tissue of N + OC-exposed adolescent rats, however this decrease was not seen in the adult rats given the same N + OC exposure. This finding of decreased levels of the ketone body BHBA suggests that exposure to N + OC may lead to an energetic shift to fat metabolism in an age-dependent manner. Although the brain is capable of metabolizing fatty acids, they are a poor fuel compared to glucose in regard to energy production and come with a few disadvantages [34,35]. Fatty acids are used to derive cellular energy via the process of β-oxidation in the mitochondria. One of the byproducts of fatty acid β-oxidation is the generation of superoxide, which is responsible for creating a cellular state of oxidative stress [36]. Furthermore, the production of superoxide in the brain is especially troublesome due to poor neuronal anti-oxidative defense, as there are lesser antioxidant enzymes in the brain as compared with other organs [37]. Interestingly, it has also been determined that N + OC exposure leads to greater levels of reactive oxygen species (ROS) production in the brains of rats [16]. This increase in ROS could be due to the observed loss of complex IV activity in N + OC-exposed rats, which allows for the escape of electrons from complex III during oxidative phosphorylation [16]. Another problem associated with deriving energy via fatty acids and β-oxidation in the brain is that, in order to generate a set amount of ATP, generation via a fatty acid will require more oxygen than if generating that same amount of ATP via glucose. This means that there would be an increased risk for neurons to come into contact with a hypoxic environment. A previous study showed that exposure to N + OC reduced local cerebral perfusion, which may also contribute to a reduced availability of oxygen in the brain [31]. Additionally, ATP is produced at a slower rate when derived via fatty acids as compared to blood glucose, which can be a detriment in times of extended neuronal firing [38]. These reasons demonstrate both why it is a disadvantage to use fatty acids as fuel for the brain and an N + OC induced shift to fat metabolism may be responsible for the observed stroke severity in rats (Figure 9).

Along with cell membranes, PC are crucial for the synthesis of the synaptic vesicles (SV) in neurons, and accumulation of phospholipids due to N + OC exposure is indicative of vesicular defects. Aberrations in PC levels could result in faults in SV synthesis, SV fusion with the synaptic membrane, or SV recycling, in all of which phospholipids play an important role [39]. A further look into synaptic changes that could be caused by alterations in synaptic vesicle composition is found in a study that observed significantly decreased post-tetanic potentiation and paired-pulse facilitation (PPF), a simple form of presynaptic plasticity that involves a transient increase in the probability of SV release, in nicotine-exposed female rats compared to the saline group [32]. The findings of the study showed nicotine disrupted 17β-estradiol (E2)-mediated phosphorylation of post-synaptic NMDA receptor subunit NR2B. This effect on the post-synaptic density was paired with a decrease in E2 receptor subtype beta (ER-β) levels following either nicotine or N + OC exposure [16,32]. Synaptic proteins such as NR2B and ER-β require palmitoylation for membrane localization/trafficking and signaling [40,41,42,43,44,45]. Palmitoylation is an enzymatic post-translational modification in which a fatty acid palmitoyl chain is reversibly attached to a cysteine residue on a protein [46,47]. The availability of fatty acid metabolites has been linked to both increases and decreases in palmitate synthesis and subsequent attachment of the palmitoyl chain to biomolecules [43]. It has been observed that palmitoylation of human estrogen receptors is crucial for receptor interaction with the protein caveolin-1 and its subsequent localization to the plasma membrane [48]. When the physical interaction between estrogen receptors and caveolin-1 is disrupted, membrane estrogen effects are eliminated [49,50], suggesting that palmitoylation is a crucial modification for membrane translocation of estrogen receptors. Therefore, N + OC-induced metabolic changes in fatty acids cause an imbalance in the process of NR2B and ER-β palmitoylation, and thus reduce synaptic or membrane signaling [14,16,32].

To corroborate the metabolomic findings, we investigated steady-state protein levels of key enzymes in the fatty acid metabolism pathway, viz., CPT1, CPT2, ACSL, FASN, and MCD, and we did not observe significant changes in protein levels of these enzymes. In fact, all the enzymes under investigation require a form of post-translational modification. For example, both CPT1 and CPT2 require phosphorylation, acetylation, or nitration while ACSL requires acetylation and phosphorylation to execute its enzyme activity [51,52]. Although the current study does not present data showing post-translational modification status or level of enzyme activity, the fact that N + OC significantly changes glycolysis, TCA cycle, and fat metabolism in the brain constitutes a troubling basis for future neurological disease development in women smokers who also use OC. Apart from worsened ischemic damage and outcome after stroke [5], OC has been implicated in the development of major depressive disorder and anxiety [8,9,10]. Additionally, the current findings that N + OC alters cerebral lipid metabolism may have a wider implication as similar changes might occur in other organs/tissue and aberrations in lipid homeostasis are suggested to be the underlying cause of metabolic diseases such as obesity, type 2 diabetes mellitus (T2D), non-alcoholic fatty liver disease, hypertriglyceridemia, and hyperlipidemia, which often result in metabolic syndrome [53]. One potential therapeutic avenue to improve the aberrations of fatty acid metabolism is the use of fibrates, which are approved by the Food and Drug Administration [54]. Fibrates are a class of drugs traditionally used for the treatment of dyslipidemia, an imbalance of lipids in the blood, and their therapeutic effects are mediated via the transcription of genes that encode lipoprotein metabolism mediating proteins [4,55]. An in vitro study shows that the fibrate-class drug bezafibrate possibly stimulated the gene expression of enzymes that facilitate the β-oxidation pathway, signifying the ability of fibrates to correct fatty oxidation defects [56,57]. Future studies need to be conducted to determine whether N + OC-induced accumulation of fatty acids can be resolved via treatment with fibrates.

## Data Availability

The data that support the findings of this study are available upon request to the corresponding author.

## Supplementary Materials

The following supporting information can be downloaded at: https://www.mdpi.com/article/10.3390/ijms232416075/s1, Table S1a: Fatty Acid Synthesis and Long Chain Fatty Acid metabolites in the Cortex of Adolescent Female Rats; Table S1b: Fatty Acid Synthesis and Long Chain Fatty Acid metabolites in the Cortex of Adult Female Rats; Table S2a: Branched Fatty Acid and Dicarboxylate Metabolites in the Cortex of Adolescent Female Rats; Table S2b: Branched Fatty Acid, Dicarboxylate, Amino and Metabolism in the Cortex of Adult Female Rats; Table S3a: Acyl Carnitine Metabolism in the Cortex of Adolescent Female Rats; Table S3b: Acyl Carnitine Metabolism in the Cortex of Adult Female Rats; Table S4a: Carnitine Metabolism, Ketone Bodies, Acetylcholine, Acyl Choline, and Monohydroxy Metabolites in the Cortex of Adolescent Female Rats; Table S4b: Carnitine Metabolism, Ketone Bodies, Acetylcholine, Acyl Choline, and Monohydroxy Metabolites in the Cortex of Adult Female Rats; Table S5a: Phosphatidylcholine (PC) Concentrations Metabolites in the Cortex of Adolescent Female Rats; Table S5b: Phosphatidylcholine (PC) Concentrations Metabolites in the Cortex of Adult Female Rats.
