# Supplementary material for: The Impact of Nicotine along with Oral Contraceptive Exposure on Brain Fatty Acid Metabolism in Female Rats
Source: Int J Mol Sci. 2022 Dec 16;23(24):16075. doi: 10.3390/ijms232416075 (PMC9780830; doi:10.3390/ijms232416075)
Supplement: Supplementary file 1 [file ijms-23-16075-s001.zip › Supplementary material/ijms-1962720-Supplementary Tables.pdf]

Supplementary Tables:

Supplementary Table S1a. Fatty Acid Synthesis and Long Chain Fatty Acid metabolites in the Cortex of Adolescent Female Rats

Metabolites	Nicotine (N) /saline (S)			Oral contraceptives (OC)/S			N + OC / S			N + OC / N			N + OC / OC		
	N/S	p-value	q-value	OC/S	p-value	q-value	N/+OC S	p-value	q-value	N+OC/N	p-value	q-value	N+OC/OC	p-value	q-value
malonylcarnitine	**1.18**	0.0537	0.2123	1.10	0.3439	0.5969	**1.25**	0.0139	0.0544	1.05	0.5723	0.9995	1.14	0.1188	0.4562
malonate	0.99	0.7765	0.7410	0.94	0.5120	0.6719	**0.84**	0.0608	0.1441	0.85	0.1091	0.8704	0.90	0.2152	0.5268
acetyl CoA	1.19	0.1787	0.3754	1.08	0.7988	0.7438	1.18	0.3096	0.3929	0.99	0.7376	0.9996	1.09	0.4450	0.6754
myristate (14:0)	1.02	0.7515	0.7329	1.05	0.2837	0.5779	1.00	0.8975	0.7187	0.98	0.6561	0.9996	0.95	0.2307	0.5268
palmitate (16:0)	**1.17**	0.0663	0.2190	**1.17**	0.0710	0.3425	1.14	0.1242	0.2407	0.98	0.7562	0.9996	0.97	0.7807	0.7656
palmitoleate (16:1n7)	1.07	0.4030	0.5581	0.96	0.6297	0.7094	1.11	0.2031	0.3162	1.04	0.6581	0.9996	**1.16**	0.0817	0.4035
margarate (17:0)	**1.14**	0.0219	0.1273	**1.17**	0.0110	0.1397	**1.20**	0.0032	0.0218	1.05	0.4733	0.9444	1.03	0.6548	0.7439
10-heptadecenoate (17:1n7)	1.10	0.4158	0.5700	0.99	0.9489	0.7685	1.14	0.2200	0.3347	1.04	0.6757	0.9996	1.15	0.1973	0.5120
stearate (18:0)	**1.17**	0.0684	0.2207	**1.30**	0.0089	0.1314	**1.22**	0.0575	0.1420	1.04	0.9351	0.9996	0.94	0.4435	0.6754
oleate/vaccenate (18:1)	1.16	0.1937	0.3858	1.09	0.4676	0.6443	1.14	0.1959	0.3108	0.98	0.9949	0.9996	1.05	0.5658	0.7078
nonadecanoate (19:0)	1.06	0.4956	0.6188	**1.16**	0.0631	0.3186	1.05	0.7108	0.6646	0.99	0.7552	0.9996	0.90	0.1333	0.4699
10-nonadecenoate (19:1n9)	**1.18**	0.0687	0.2207	1.12	0.2144	0.5434	1.09	0.2710	0.3720	0.92	0.4596	0.9444	0.97	0.8860	0.7872
arachidate (20:0)	1.15	0.2625	0.4375	**1.38**	0.0099	0.1323	1.08	0.5875	0.6005	0.94	0.5600	0.9936	**0.78**	0.0379	0.2704
eicosenoate (20:1)	1.09	0.2411	0.4197	1.06	0.4719	0.6449	1.00	0.7745	0.6792	0.92	0.3736	0.9096	0.94	0.6642	0.7439
erucate (22:1n9)	1.22	0.1532	0.3446	**1.29**	0.0794	0.3627	0.98	0.8367	0.7034	0.80	0.2198	0.8760	0.76	0.1199	0.4562

Supplementary Table S1b. Fatty Acid Synthesis and Long Chain Fatty Acid metabolites in the Cortex of Adult Female Rats

Metabolites	Nicotine (N) /saline (S)			Oral contraceptives (OC)/S			N + OC / S			N + OC / N			N + OC / OC		
	N/S	p-value	q-value	OC/S	p-value	q-value	N/+OC S	p-value	q-value	N+OC/N	p-value	q-value	N+OC/OC	p-value	q-value
malonylcarnitine	**1.16**	0.0853	0.4469	1.09	0.3035	0.8021	**1.15**	0.0980	0.2265	0.99	0.9449	0.6519	1.02	0.5222	0.5102
malonate	1.04	0.5900	0.7530	0.87	0.1490	0.5897	1.06	0.4758	0.4815	1.02	0.8610	0.6356	**1.23**	0.0334	0.1100
acetyl CoA	0.84	0.2245	0.5988	0.97	0.8298	0.9974	1.21	0.1907	0.3143	**1.44**	0.0134	0.0898	**1.39**	0.1291	0.2543
myristate (14:0)	1.04	0.4268	0.6792	1.07	0.1213	0.5255	**1.11**	0.0268	0.1177	1.07	0.1460	0.2831	1.06	0.4859	0.4921
palmitate (16:0)	1.04	0.7985	0.7880	**1.20**	0.0581	0.4320	1.17	0.1076	0.2378	1.12	0.1735	0.3087	0.98	0.7660	0.5919
palmitoleate (16:1n7)	0.92	0.3143	0.6390	0.96	0.5892	0.9562	1.09	0.5529	0.5113	1.19	0.1125	0.2601	0.99	0.2591	0.3587
margarate (17:0)	1.01	0.9430	0.8286	**1.16**	0.0186	0.2887	**1.19**	0.0058	0.0534	**1.18**	0.0071	0.0728	1.05	0.6603	0.5714
10-heptadecenoate (17:1n7)	0.97	0.7989	0.7880	0.95	0.6700	0.9705	1.29	0.1961	0.3143	1.33	0.1234	0.2673	1.27	0.0879	0.2173
stearate (18:0)	1.08	0.5498	0.7495	**1.23**	0.0418	0.4062	**1.28**	0.0134	0.0791	**1.19**	0.0559	0.1850	1.03	0.6393	0.5671
oleate/vaccenate (18:1)	1.03	0.8940	0.8133	1.17	0.2521	0.7492	1.03	0.9699	0.6487	1.00	0.9238	0.6498	0.86	0.2678	0.3663
nonadecanoate (19:0)	1.10	0.2628	0.6093	0.99	0.7696	0.9974	**1.16**	0.0723	0.2037	1.06	0.4866	0.5052	**1.30**	0.0379	0.1201
10-nonadecenoate (19:1n9)	1.01	0.9550	0.8286	1.08	0.4193	0.8624	1.10	0.4143	0.4615	1.09	0.4470	0.4886	1.19	0.9930	0.6453
arachidate (20:0)	1.16	0.1713	0.5510	1.15	0.2485	0.7470	1.08	0.4377	0.4648	0.93	0.5482	0.5228	**1.20**	0.7021	0.5794
eicosenoate (20:1)	1.00	0.9832	0.8286	**1.19**	0.0955	0.5202	1.03	0.9085	0.6399	1.03	0.8919	0.6438	1.00	0.1197	0.2495
erucate (22:1n9)	0.99	0.9724	0.8286	1.25	0.1803	0.6510	1.15	0.5305	0.5089	1.16	0.5082	0.5113	1.14	0.4712	0.4869

Supplementary Table S2a. Branched Fatty Acid and Dicarboxylate Metabolites in the Cortex of Adolescent Female Rats

Metabolites	Nicotine (N) /saline (S)			Oral contraceptives (OC)/S			N + OC / S			N + OC / N			N + OC / OC		
	N/S	p-value	q-value	OC/S	p-value	q-value	N/+OC S	p-value	q-value	N+OC/N	p-value	q-value	N+OC/OC	p-value	q-value
15-methylpalmitate (i17:0)	1.14	0.2041	0.3907	**1.35**	0.0046	0.0982	**1.36**	0.0033	0.0218	**1.20**	0.0792	0.8064	1.01	0.9059	0.7882
17-methylstearate (i19:0)	1.04	0.6029	0.6677	1.13	0.1783	0.5034	1.10	0.2835	0.3790	1.05	0.5779	0.9995	0.97	0.7802	0.7656
dimethylmalonic acid	1.22	0.3405	0.5058	1.01	0.8424	0.7447	0.90	0.6954	0.6610	0.74	0.1809	0.8704	0.89	0.5554	0.7020
glutarate (C5-DC)	0.83	0.2065	0.3933	0.80	0.1355	0.4374	**0.74**	0.0245	0.0854	0.90	0.3059	0.8898	0.92	0.4288	0.6672
2-hydroxyglutarate	**1.25**	0.0816	0.2360	1.02	0.6288	0.7094	1.02	0.7509	0.6792	0.82	0.1515	0.8704	1.00	0.8679	0.7834
2-hydroxyadipate	**0.65**	0.0505	0.2051	0.90	0.6835	0.7293	0.70	0.1087	0.2227	1.07	0.7136	0.9996	0.77	0.2274	0.5268
3-hydroxyadipate*	0.78	0.1935	0.3858	0.98	0.7248	0.7358	0.91	0.4136	0.4772	1.17	0.6244	0.9996	0.93	0.6402	0.7399
maleate	0.89	0.2148	0.4002	0.98	0.8446	0.7447	1.02	0.8589	0.7075	1.14	0.1573	0.8704	1.04	0.7087	0.7586
2-aminoheptanoate	1.04	0.5214	0.6274	0.99	0.7971	0.7438	1.48	0.2525	0.3601	**1.43**	0.0770	0.8064	1.50	0.1628	0.4946
propionylcarnitine (C3)	**1.51**	0.0090	0.0649	**1.58**	0.0042	0.0982	**1.64**	0.0033	0.0218	1.09	0.7135	0.9996	1.04	0.9288	0.7902
methylmalonate (MMA)	0.95	0.6693	0.6981	1.09	0.3567	0.5981	0.95	0.6657	0.6443	0.99	0.9961	0.9996	0.87	0.1781	0.4946

Supplementary Table S2b. Branched Fatty Acid, Dicarboxylate, Amino and Metabolism in the Cortex of Adult Female Rats

Metabolites	Nicotine (N) /saline (S)			Oral contraceptives (OC)/S			N + OC / S			N + OC / N			N + OC / OC		
	N/S	p-value	q-value	OC/S	p-value	q-value	N/+OC S	p-value	q-value	N+OC/N	p-value	q-value	N+OC/OC	p-value	q-value
15-methylpalmitate (i17:0)	1.12	0.2460	0.6063	1.12	0.1968	0.6723	**1.39**	0.0092	0.0703	1.24	0.1324	0.2730	1.07	0.1691	0.2878
17-methylstearate (i19:0)	1.00	0.8373	0.7973	1.03	0.6312	0.9597	**1.17**	0.0525	0.1661	**1.17**	0.0814	0.2174	1.08	0.1398	0.2630
dimethylmalonic acid	0.90	0.5553	0.7495	0.90	0.4925	0.8991	0.80	0.2087	0.3201	0.89	0.5004	0.5106	1.13	0.5636	0.5289
glutarate (C5-DC)	0.88	0.4406	0.6892	0.91	0.5040	0.9000	0.92	0.5373	0.5111	1.05	0.8766	0.6394	1.03	0.9588	0.6387
2-hydroxyglutarate	0.89	0.4700	0.6998	0.88	0.5157	0.9061	0.97	0.9459	0.6454	1.09	0.4296	0.4825	1.03	0.4731	0.4869
2-hydroxyadipate	1.10	0.2418	0.6036	0.67	0.1911	0.6610	0.87	0.9416	0.6442	0.79	0.2720	0.3840	1.34	0.1679	0.2878
3-hydroxyadipate*	0.88	0.5365	0.7494	0.93	0.8289	0.9974	1.02	0.9965	0.6551	1.15	0.5394	0.5209	0.93	0.8323	0.5998
maleate	**0.83**	0.0878	0.4469	0.84	0.1082	0.5255	0.87	0.2217	0.3291	1.05	0.6177	0.5564	**0.79**	0.6932	0.5761
2-aminoheptanoate	1.56	0.1163	0.4742	**0.64**	0.0828	0.5019	**2.27**	0.0041	0.0430	1.45	0.1664	0.3049	1.20	0.0000	0.0004
propionylcarnitine (C3)	0.81	0.1312	0.4937	1.05	0.9110	0.9974	0.90	0.3238	0.4050	1.10	0.5938	0.5428	1.15	0.2727	0.3695
methylmalonate (MMA)	1.02	0.6903	0.7747	1.08	0.3952	0.8538	0.97	0.7657	0.5799	0.95	0.4869	0.5052	0.93	0.2525	0.3587

Supplementary Table S3a. Acyl Carnitine Metabolism in the Cortex of Adolescent Female Rats

Metabolites	Nicotine (N) /saline (S)			Oral contraceptives (OC)/S			N + OC / S			N + OC / N			N + OC / OC		
	N/S	p-value	q-value	OC/S	p-value	q-value	N/+OC S	p-value	q-value	N+OC/N	p-value	q-value	N+OC/OC	p-value	q-value
acetylcarnitine (C2)	**1.20**	0.0554	0.2144	1.13	0.2069	0.5379	**1.26**	0.0159	0.0600	1.05	0.5977	0.9996	1.11	0.2314	0.5268
3-hydroxybutyrylcarnitine (1)	1.05	0.7237	0.7256	0.86	0.2639	0.5670	1.02	0.9931	0.7389	0.97	0.7303	0.9996	1.18	0.2603	0.5515
3-hydroxybutyrylcarnitine (2)	1.02	0.6999	0.7167	0.95	0.8115	0.7438	1.24	0.2404	0.3500	1.22	0.4276	0.9423	1.30	0.1593	0.4946
laurylcarnitine (C12)	0.97	0.9741	0.7724	1.14	0.4628	0.6427	1.14	0.3452	0.4253	1.18	0.3291	0.8898	1.00	0.8324	0.7751
myristoylcarnitine (C14)	0.98	0.9376	0.7635	1.10	0.4235	0.6161	1.15	0.2244	0.3388	1.17	0.2550	0.8871	1.04	0.6745	0.7439
palmitoylcarnitine (C16)	1.09	0.5804	0.6564	1.11	0.4293	0.6224	**1.38**	0.0188	0.0697	**1.27**	0.0676	0.8064	1.24	0.1101	0.4562
palmitoleoylcarnitine (C16:1)*	1.06	0.7830	0.7430	1.12	0.5343	0.6764	**1.37**	0.0951	0.1980	1.29	0.1608	0.8704	1.22	0.2881	0.5746
stearoylcarnitine (C18)	**1.26**	0.0641	0.2190	**1.39**	0.0073	0.1303	**1.44**	0.0035	0.0228	1.14	0.2517	0.8871	1.04	0.7942	0.7675
linoleoylcarnitine (C18:2)*	1.22	0.2224	0.4066	1.12	0.4782	0.6474	**1.51**	0.0102	0.0452	1.24	0.1596	0.8704	**1.35**	0.0568	0.3193
oleoylcarnitine (C18:1)	1.18	0.3165	0.4782	1.21	0.2483	0.5670	**1.39**	0.0537	0.1387	1.18	0.3414	0.8898	1.15	0.4249	0.6672
myristoleoylcarnitine (C14:1)*	1.00	0.9739	0.7724	1.13	0.7259	0.7358	1.21	0.2943	0.3859	1.21	0.3094	0.8898	1.07	0.4830	0.6915
arachidoylcarnitine (C20)*	1.25	0.1676	0.3609	0.95	0.9667	0.7685	1.01	0.9041	0.7187	0.80	0.2068	0.8713	1.06	0.8712	0.7834
arachidonoylcarnitine (C20:4)	**1.27**	0.0022	0.0241	**1.19**	0.0234	0.1907	**1.34**	0.0002	0.0034	1.06	0.4393	0.9444	1.13	0.1040	0.4562
adrenoylcarnitine (C22:4)*	**1.54**	0.0325	0.1722	0.91	0.6838	0.7293	1.24	0.2458	0.3542	0.81	0.3122	0.8898	1.36	0.1192	0.4562
dihomo-linolenoylcarnitine (C20:3n3 or 6)*	**1.31**	0.0806	0.2346	1.07	0.5584	0.6844	**1.33**	0.0613	0.1444	1.01	0.8966	0.9996	1.24	0.1918	0.5120
dihomo-linoleoylcarnitine (C20:2)*	**1.37**	0.0566	0.2144	1.00	0.8660	0.7508	**1.42**	0.0284	0.0932	1.03	0.7627	0.9996	**1.41**	0.0188	0.1988
eicosenoylcarnitine (C20:1)*	**1.28**	0.0594	0.2189	1.20	0.1349	0.4374	**1.35**	0.0288	0.0932	1.05	0.7503	0.9996	1.12	0.4703	0.6862
erucoylcarnitine (C22:1)*	1.36	0.1293	0.3182	0.96	0.9143	0.7613	0.99	0.9855	0.7360	0.73	0.1338	0.8704	1.03	0.8999	0.7882
docosahexaenoylcarnitine (C22:6)*	**1.26**	0.0408	0.1848	**1.31**	0.0189	0.1762	**1.57**	0.0003	0.0039	**1.24**	0.0746	0.8064	1.20	0.1410	0.4740
margaroylcarnitine (C17)*	1.17	0.3524	0.5199	1.09	0.4124	0.6079	**1.27**	0.0555	0.1420	1.09	0.3131	0.8898	1.17	0.2634	0.5543
pentadecanoylcarnitine (C15)*	1.04	0.8762	0.7549	1.07	0.5411	0.6827	**1.33**	0.0608	0.1441	**1.28**	0.0844	0.8302	1.24	0.1994	0.5120

Supplementary Table S3b. Acyl Carnitine Metabolism in the Cortex of Adult Female

Rats

Metabolites	Nicotine (N) /saline (S)			Oral contraceptives (OC)/S			N + OC / S			N + OC / N			N + OC / OC		
	N/S	p-value	q-value	OC/S	p-value	q-value	N/+OC S	p-value	q-value	N+OC/N	p-value	q-value	N+OC/OC	p-value	q-value
acetylcarnitine (C2)	1.14	0.1794	0.5637	0.99	0.9545	0.9974	1.16	0.1095	0.2378	1.02	0.7906	0.6119	1.19	0.0978	0.2335
3-hydroxybutyrylcarnitine (1)	1.15	0.2734	0.6199	0.68	0.0092	0.1806	1.29	0.2070	0.3201	1.12	0.8651	0.6374	1.30	0.0002	0.0038
3-hydroxybutyrylcarnitine (2)	1.00	0.7110	0.7749	0.56	0.0078	0.1650	0.74	0.2366	0.3440	0.74	0.1224	0.2668	0.84	0.1235	0.2511
laurylcarnitine (C12)	0.93	0.9491	0.8286	0.97	0.8107	0.9974	0.73	0.1330	0.2679	0.79	0.1497	0.2843	1.10	0.0829	0.2092
myristoylcarnitine (C14)	0.88	0.5780	0.7503	0.97	0.9266	0.9974	0.79	0.1931	0.3143	0.90	0.4518	0.4924	1.05	0.1641	0.2862
palmitoylcarnitine (C16)	0.94	0.9653	0.8286	0.93	0.9526	0.9974	0.83	0.3934	0.4466	0.88	0.4177	0.4749	0.94	0.4269	0.4731
palmitoleoylcarnitine (C16:1)*	0.86	0.6720	0.7747	0.88	0.9224	0.9974	0.72	0.1934	0.3143	0.83	0.3768	0.4567	0.81	0.2281	0.3367
stearoylcarnitine (C18)	1.04	0.9461	0.8286	1.09	0.4963	0.8991	1.30	0.0391	0.1423	1.24	0.0456	0.1571	1.04	0.1590	0.2827
linoleoylcarnitine (C18:2)*	1.09	0.4916	0.7142	0.99	0.9105	0.9974	1.31	0.0890	0.2167	1.20	0.3035	0.4051	1.13	0.1113	0.2376
oleoylcarnitine (C18:1)	0.92	0.9478	0.8286	0.92	0.9178	0.9974	0.77	0.3488	0.4220	0.83	0.3832	0.4602	0.82	0.2989	0.3853
myristoleoylcarnitine (C14:1)*	0.79	0.4990	0.7173	0.86	0.8785	0.9974	0.68	0.1022	0.2319	0.86	0.3307	0.4246	0.91	0.1372	0.2595
arachidoylcarnitine (C20)*	1.19	0.4123	0.6667	0.83	0.2939	0.7878	1.06	0.7543	0.5773	0.89	0.6111	0.5533	0.95	0.1749	0.2921
arachidonoylcarnitine (C20:4)	1.02	0.6787	0.7747	0.95	0.5488	0.9303	1.07	0.3607	0.4274	1.04	0.6154	0.5556	1.01	0.1329	0.2555
adrenoylcarnitine (C22:4)*	1.37	0.0813	0.4366	0.72	0.1724	0.6338	1.03	0.5815	0.5240	0.75	0.2272	0.3546	0.81	0.0578	0.1646
dihomo-linolenoylcarnitine (C20:3n3 or 6)*	1.14	0.2881	0.6242	0.75	0.1524	0.5946	1.01	0.6433	0.5354	0.89	0.5464	0.5228	0.89	0.0604	0.1679
dihomo-linoleoylcarnitine (C20:2)*	1.07	0.6028	0.7606	0.96	0.7945	0.9974	1.38	0.0460	0.1536	1.29	0.1349	0.2739	1.00	0.0251	0.0938
eicosenoylcarnitine (C20:1)*	1.02	0.8648	0.8043	0.92	0.7645	0.9974	1.03	0.6366	0.5354	1.02	0.7622	0.6057	0.95	0.4409	0.4819
erucoylcarnitine (C22:1)*	1.17	0.3242	0.6405	0.87	0.5578	0.9353	0.88	0.7013	0.5556	0.76	0.1730	0.3087	0.88	0.8390	0.6005
docosahexaenoylcarnitine (C22:6)*	1.01	0.9810	0.8286	0.97	0.6676	0.9705	1.14	0.2574	0.3596	1.13	0.2477	0.3679	1.04	0.1207	0.2495
margaroylcarnitine (C17)*	0.98	0.9840	0.8286	1.08	0.5018	0.8991	1.01	0.7304	0.5650	1.04	0.7154	0.5976	1.01	0.7429	0.5885
pentadecanoylcarnitine (C15)*	0.95	0.9103	0.8161	0.88	0.5633	0.9388	0.86	0.5494	0.5113	0.91	0.6267	0.5629	1.12	0.9835	0.6438

Supplementary Table S4a. Carnitine Metabolism, Ketone Bodies, Acetylcholine, Acyl Choline, and Monohydroxy Metabolites in the Cortex of Adolescent Female Rats

Metabolites	Nicotine (N) /saline (S)			Oral contraceptives (OC)/S			N + OC / S			N + OC / N			N + OC / OC		
	N/S	p-value	q-value	OC/S	p-value	q-value	N/+OC S	p-value	q-value	N+OC/N	p-value	q-value	N+OC/OC	p-value	q-value
deoxycarnitine	1.02	0.8501	0.7502	0.96	0.6170	0.7087	1.10	0.2913	0.3837	1.08	0.3851	0.9209	1.14	0.1225	0.4611
carnitine	1.22	0.0089	0.0649	1.08	0.3013	0.5904	1.26	0.0040	0.0254	1.03	0.7740	0.9996	1.16	0.0556	0.3193
3-hydroxybutyrate (BHBA)	0.82	0.2011	0.3887	0.61	0.0013	0.0600	0.68	0.0072	0.0352	0.82	0.1398	0.8704	1.10	0.5530	0.7020
acetylcholine	1.17	0.0484	0.2023	1.23	0.0087	0.1314	1.46	0.0000	0.0002	1.25	0.0041	0.3383	1.19	0.0254	0.2133
docosahexaenoylcholine	1.09	0.3694	0.5286	1.11	0.2703	0.5696	1.01	0.8649	0.7096	0.92	0.4660	0.9444	0.91	0.3500	0.6254
2-hydroxylignocerate*	1.49	0.0684	0.2207	1.14	0.5473	0.6827	1.71	0.0051	0.0298	1.15	0.2952	0.8898	1.50	0.0246	0.2106
2-hydroxynervonate*	1.33	0.0804	0.2346	1.56	0.0166	0.1706	1.23	0.1802	0.3036	0.93	0.6733	0.9996	0.79	0.2710	0.5599
13-HODE + 9-HODE	1.36	0.9486	0.7681	1.52	0.3252	0.5969	1.28	0.7167	0.6646	0.94	0.7653	0.9996	0.84	0.5327	0.7020

Supplementary Table S4b. Carnitine Metabolism, Ketone Bodies, Acetylcholine, Acyl Choline, and Monohydroxy Metabolites in the Cortex of Adult Female Rats

Metabolites	Nicotine (N) /saline (S)			Oral contraceptives (OC)/S			N + OC / S			N + OC / N			N + OC / OC		
	N/S	p-value	q-value	OC/S	p-value	q-value	N/+OC S	p-value	q-value	N+OC/N	p-value	q-value	N+OC/OC	p-value	q-value
deoxycarnitine	0.94	0.5563	0.7495	0.97	0.7241	0.9974	0.96	0.6417	0.5354	1.02	0.9018	0.6438	0.82	0.9103	0.6227
carnitine	1.09	0.2206	0.5988	0.92	0.3192	0.8060	1.13	0.0959	0.2258	1.03	0.6509	0.5750	1.02	0.0092	0.0493
3-hydroxybutyrate (BHBA)	1.03	0.7932	0.7878	0.65	0.0037	0.1261	1.27	0.2784	0.3816	1.24	0.4095	0.4695	1.68	0.0001	0.0026
acetylcholine	1.07	0.3867	0.6503	1.08	0.3537	0.8437	1.36	0.0001	0.0033	1.28	0.0020	0.0369	1.27	0.0024	0.0186
docosahexaenoylcholine	0.90	0.5085	0.7254	1.08	0.4056	0.8565	0.85	0.1876	0.3143	0.94	0.5065	0.5112	1.11	0.0341	0.1109
2-hydroxylignocerate*	1.09	0.5788	0.7503	1.70	0.0179	0.2887	0.98	0.9520	0.6461	0.90	0.5384	0.5209	0.53	0.0154	0.0688
2-hydroxynervonate*	1.12	0.6219	0.7616	1.30	0.1881	0.6547	1.23	0.3963	0.4478	1.10	0.7209	0.5983	1.04	0.6348	0.5671
13-HODE + 9-HODE	1.16	0.7652	0.7749	1.01	0.9535	0.9974	0.90	0.9667	0.6487	0.78	0.7972	0.6131	0.71	0.9868	0.6440

Supplementary Table S5a. Phosphatidylcholine (PC) Concentrations Metabolites in the Cortex of Adolescent Female Rats

Metabolites	Nicotine (N) /saline (S)			Oral contraceptives (OC)/S			N + OC / S			N + OC / N			N + OC / OC		
	N/S	p-value	q-value	OC/S	p-value	q-value	N/+OC S	p-value	q-value	N+OC/N	p-value	q-value	N+OC/OC	p-value	q-value
1-myristoyl-2-palmitoyl-GPC (14:0/16:0)	1.08	0.4072	0.5622	1.05	0.6514	0.7123	1.13	0.1839	0.3036	1.05	0.6119	0.9996	1.07	0.3768	0.6437
1-myristoyl-2-arachidonoyl-GPC (14:0/20:4)*	1.27	0.0379	0.1754	1.01	0.7183	0.7358	1.15	0.1243	0.2407	0.91	0.5735	0.9995	1.14	0.2360	0.5296
1,2-dipalmitoyl-GPC (16:0/16:0)	1.00	0.9218	0.7628	1.04	0.6439	0.7094	1.11	0.1910	0.3055	1.10	0.2257	0.8784	1.07	0.3941	0.6526
1-palmitoyl-2-palmitoleoyl-GPC (16:0/16:1)*	1.11	0.2122	0.3974	1.08	0.3372	0.5969	1.24	0.0162	0.0605	1.13	0.2284	0.8784	1.15	0.1362	0.4715
1-palmitoyl-2-stearoyl-GPC (16:0/18:0)	1.01	0.9359	0.7635	1.04	0.8609	0.7479	1.07	0.6309	0.6255	1.06	0.6889	0.9996	1.03	0.7599	0.7656
1-palmitoyl-2-oleoyl-GPC (16:0/18:1)	1.07	0.2313	0.4066	1.13	0.0303	0.2097	1.17	0.0083	0.0394	1.09	0.1329	0.8704	1.03	0.6103	0.7328
1-palmitoyl-2-linoleoyl-GPC (16:0/18:2)	1.22	0.0456	0.1999	1.16	0.1748	0.4968	1.35	0.0043	0.0261	1.10	0.3545	0.8917	1.16	0.1145	0.4562
1-palmitoyl-2-dihomo-linolenoyl-GPC (16:0/20:3n3 or 6)*	0.92	0.5085	0.6253	1.04	0.8760	0.7515	0.94	0.5119	0.5493	1.02	0.9957	0.9996	0.91	0.4175	0.6672
1-palmitoyl-2-arachidonoyl-GPC (16:0/20:4n6)	1.12	0.0648	0.2190	1.07	0.2387	0.5634	1.14	0.0263	0.0898	1.02	0.6917	0.9996	1.07	0.2798	0.5732
1-palmitoyl-2-docosahexaenoyl-GPC (16:0/22:6)	1.12	0.1651	0.3591	1.19	0.0293	0.2097	1.24	0.0084	0.0394	1.11	0.1904	0.8704	1.04	0.6227	0.7351
1-stearoyl-2-oleoyl-GPC (18:0/18:1)	1.02	0.7250	0.7256	1.18	0.1945	0.5217	1.07	0.4809	0.5300	1.04	0.7232	0.9996	0.90	0.5486	0.7020
1-stearoyl-2-arachidonoyl-GPC (18:0/20:4)	1.06	0.4895	0.6188	1.06	0.5681	0.6872	1.13	0.1588	0.2780	1.07	0.4670	0.9444	1.07	0.3969	0.6526
1-stearoyl-2-docosahexaenoyl-GPC (18:0/22:6)	1.17	0.2389	0.4183	1.23	0.0984	0.3843	1.25	0.0809	0.1764	1.07	0.5596	0.9936	1.02	0.9231	0.7902
1-oleoyl-2-linoleoyl-GPC (18:1/18:2)*	1.20	0.0827	0.2376	1.17	0.1282	0.4266	1.37	0.0024	0.0191	1.15	0.1648	0.8704	1.17	0.1086	0.4562
1-oleoyl-2-docosahexaenoyl-GPC (18:1/22:6)*	1.13	0.1621	0.3558	1.26	0.0078	0.1314	1.20	0.0327	0.0987	1.07	0.4423	0.9444	0.95	0.5720	0.7078
1,2-dilinoleoyl-GPC (18:2/18:2)	1.18	0.3098	0.4782	1.01	0.8563	0.7470	1.18	0.2548	0.3608	1.00	0.9005	0.9996	1.17	0.1881	0.5120
1-linoleoyl-2-arachidonoyl-GPC (18:2/20:4n6)*	1.19	0.1432	0.3335	1.09	0.4365	0.6266	1.18	0.1470	0.2606	0.99	0.9887	0.9996	1.08	0.4950	0.6934

Supplementary Table S5b. Phosphatidylcholine (PC) Concentrations Metabolites in the Cortex of Adult Female Rats

Metabolites	Nicotine (N) /saline (S)			Oral contraceptives (OC)/S			N + OC / S			N + OC / N			N + OC / OC		
	N/S	p-value	q-value	OC/S	p-value	q-value	N/+OC S	p-value	q-value	N+OC/N	p-value	q-value	N+OC/OC	p-value	q-value
1-myristoyl-2-palmitoyl-GPC (14:0/16:0)	0.95	0.6436	0.7735	1.07	0.3708	0.8437	1.08	0.3735	0.4369	1.14	0.1785	0.3132	0.89	0.9960	0.6453
1-myristoyl-2-arachidonoyl-GPC (14:0/20:4)*	1.13	0.3186	0.6405	1.12	0.2823	0.7878	1.39	0.0083	0.0676	1.23	0.0887	0.2253	0.96	0.1039	0.2369
1,2-dipalmitoyl-GPC (16:0/16:0)	0.92	0.4356	0.6837	0.97	0.9330	0.9974	1.06	0.3193	0.4040	1.15	0.0788	0.2152	0.99	0.2807	0.3739
1-palmitoyl-2-palmitoleoyl-GPC (16:0/16:1)*	0.95	0.6174	0.7613	1.02	0.7195	0.9942	1.17	0.1055	0.2347	1.23	0.0361	0.1507	0.97	0.2043	0.3186
1-palmitoyl-2-stearoyl-GPC (16:0/18:0)	0.78	0.0503	0.4037	0.92	0.6341	0.9597	0.98	0.9554	0.6461	1.26	0.0444	0.1543	0.93	0.5949	0.5426
1-palmitoyl-2-oleoyl-GPC (16:0/18:1)	0.96	0.4283	0.6792	1.01	0.7396	0.9974	1.11	0.0617	0.1870	1.16	0.0090	0.0795	1.06	0.1215	0.2498
1-palmitoyl-2-linoleoyl-GPC (16:0/18:2)	0.98	0.8380	0.7973	1.02	0.6965	0.9862	1.50	0.0002	0.0055	1.53	0.0001	0.0077	1.27	0.0009	0.0094
1-palmitoyl-2-dihomo-linolenoyl-GPC (16:0/20:3n3 or 6)*	0.77	0.0958	0.4634	0.92	0.8899	0.9974	1.42	0.0108	0.0763	1.84	0.0001	0.0045	1.64	0.0074	0.0420
1-palmitoyl-2-arachidonoyl-GPC (16:0/20:4n6)	0.97	0.5675	0.7503	1.03	0.6335	0.9597	1.15	0.0221	0.1034	1.18	0.0049	0.0600	1.03	0.0661	0.1772
1-palmitoyl-2-docosahexaenoyl-GPC (16:0/22:6)	0.99	0.8722	0.8043	1.07	0.3556	0.8437	1.22	0.0161	0.0892	1.23	0.0106	0.0832	1.07	0.1266	0.2531
1-stearoyl-2-oleoyl-GPC (18:0/18:1)	0.80	0.0525	0.4037	1.00	0.9573	0.9974	0.96	0.6324	0.5354	1.19	0.1392	0.2757	1.07	0.5949	0.5426
1-stearoyl-2-arachidonoyl-GPC (18:0/20:4)	0.86	0.0891	0.4469	1.01	0.8632	0.9974	1.09	0.3259	0.4050	1.26	0.0086	0.0795	1.01	0.4168	0.4718
1-stearoyl-2-docosahexaenoyl-GPC (18:0/22:6)	0.88	0.2709	0.6199	1.01	0.7777	0.9974	1.21	0.0941	0.2233	1.37	0.0067	0.0728	1.20	0.1614	0.2856
1-oleoyl-2-linoleoyl-GPC (18:1/18:2)*	0.93	0.5718	0.7503	1.07	0.4472	0.8816	1.44	0.0009	0.0166	1.54	0.0002	0.0081	1.14	0.0085	0.0468
1-oleoyl-2-docosahexaenoyl-GPC (18:1/22:6)*	0.96	0.6513	0.7747	1.09	0.2664	0.7631	1.24	0.0103	0.0756	1.28	0.0029	0.0492	1.14	0.1308	0.2555
1,2-dilinoleoyl-GPC (18:2/18:2)	1.02	0.9059	0.8161	1.01	0.9093	0.9974	1.41	0.0185	0.0941	1.38	0.0247	0.1263	0.97	0.0244	0.0934
1-linoleoyl-2-arachidonoyl-GPC (18:2/20:4n6)*	1.05	0.8683	0.8043	1.10	0.3779	0.8437	1.69	0.0001	0.0029	1.61	0.0002	0.0085	1.27	0.0018	0.0158

Supplementary Tables S1-5: The tables shows fold-change of the specified metabolites in the cortexes of (a) adolescent and (b) adult rats; the red colored boxes represent a significant increase ($p \leq 0.05$) in that metabolite, the pink boxes represent a narrowly missed statistical cutoff for significant increase $0.05 < p < 0.10$, the green colored boxes represent a significant decrease ($p \leq 0.05$) in that metabolite, and the light green colored boxes represent a narrowly missed statistical cutoff for significant decrease in that metabolite $0.05 < p < 0.10$. "Biochemical name*" indicates a compound that has not been confirmed based on a standard, but Metabolon is confident in its identity.
