# Supplementary material for: The Impact of Nicotine along with Oral Contraceptive Exposure on Brain Fatty Acid Metabolism in Female Rats
Source: Int J Mol Sci. 2022 Dec 16;23(24):16075. doi: 10.3390/ijms232416075 (PMC9780830; doi:10.3390/ijms232416075)

Supporting Information File:

Western Blots

Adolescent cortex (AC) - AC1

- 8 gels/blots
  - 4 normal run time
  - 4 extended run/separation
- Same loading
- Homogenate from rt. cortex

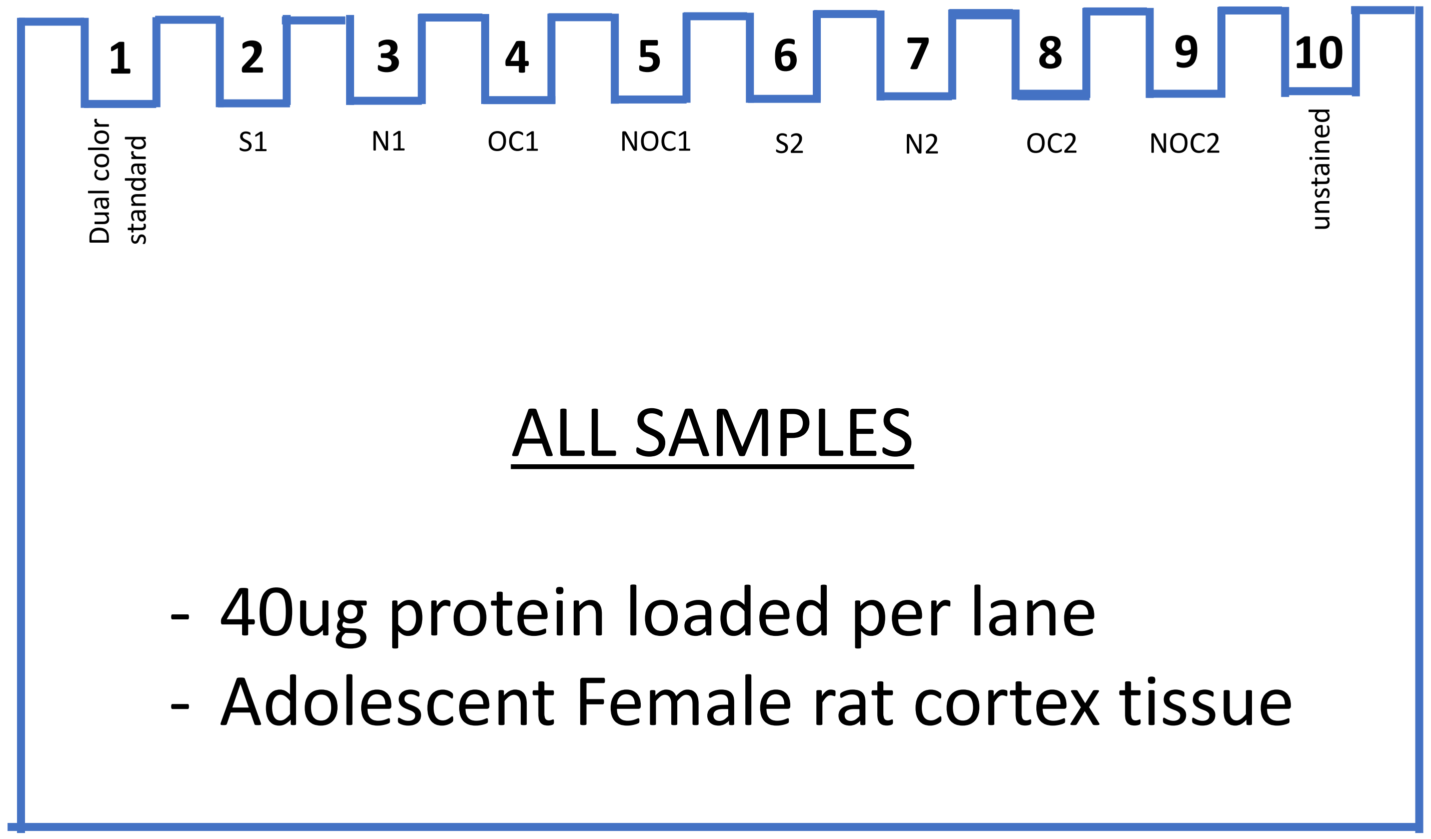

AC1

AC1F top

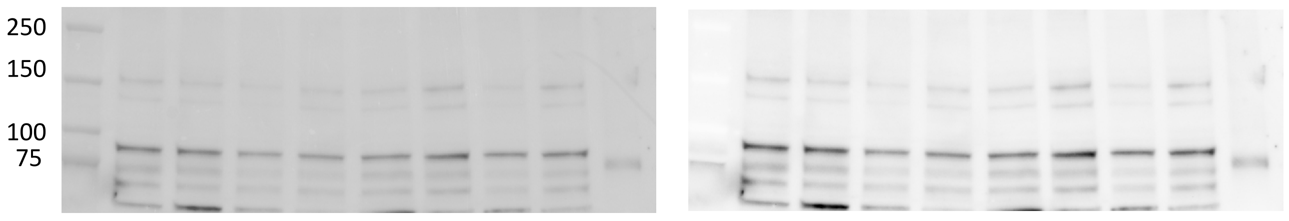

CPT 1 – 78 kDa

AC1G top

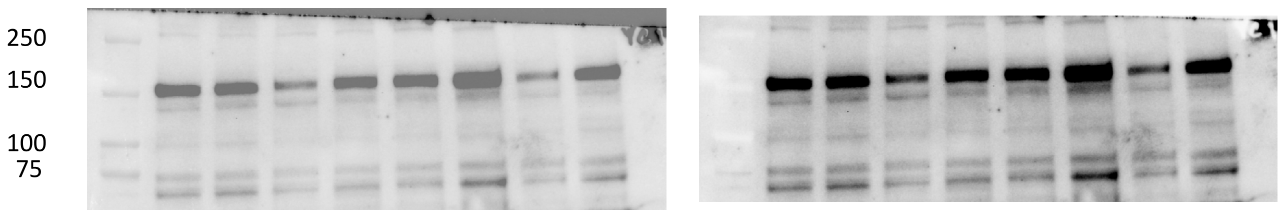

CPT 2 – 68 kDa

AC1G

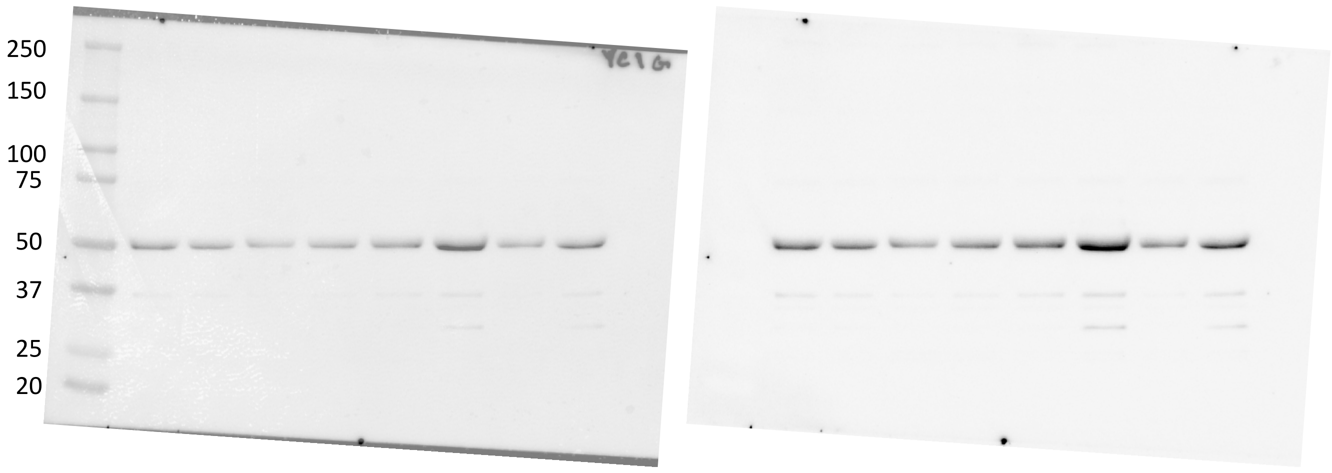

CKα – 52 kDa

AC1H

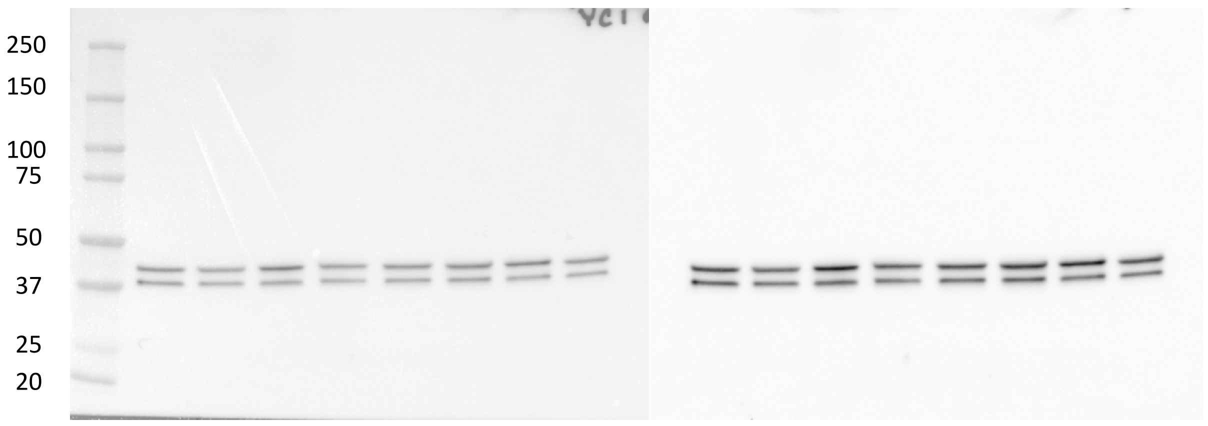

MAPK – 42,44 kDa

AC1C bottom

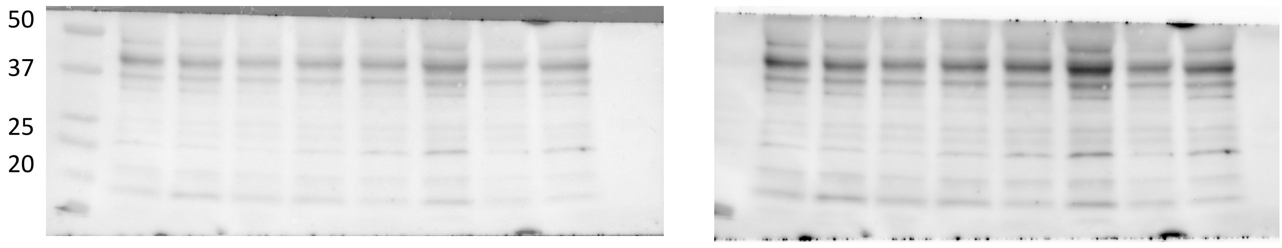

CCTβ (PCYT1B) – 42 kDa

AC1 – R1

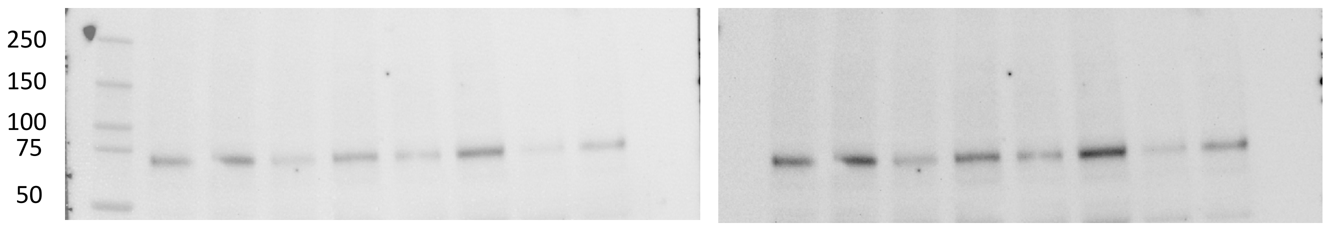

ACSL – 78 kDa

AC1 – R1

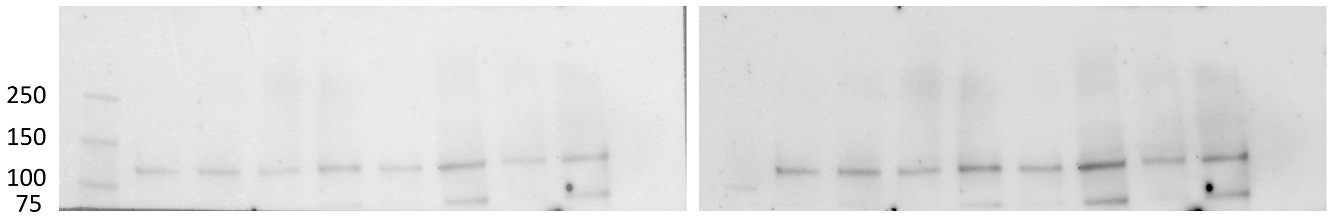

ACL – 125 kDa

AC1 – R1

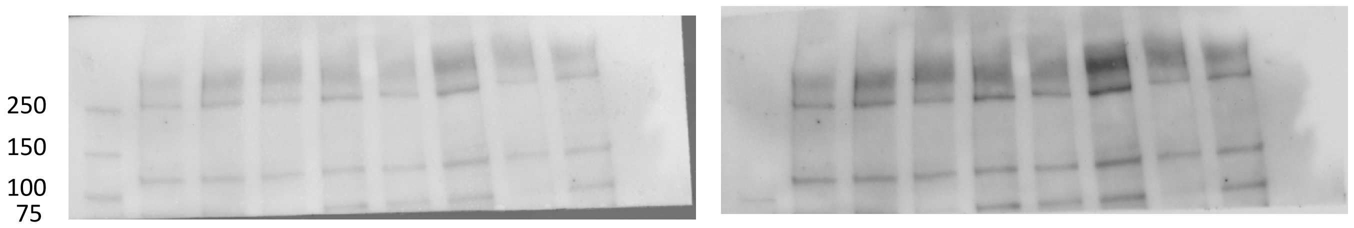

FASN – 273 kDa

AC1 – β-actin Blots

AC1F

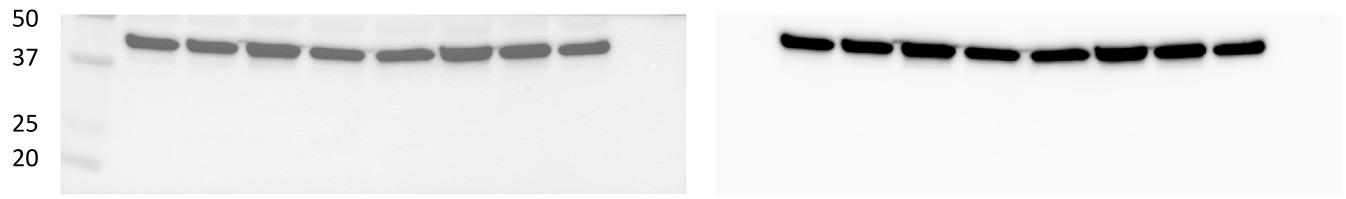

AC1C

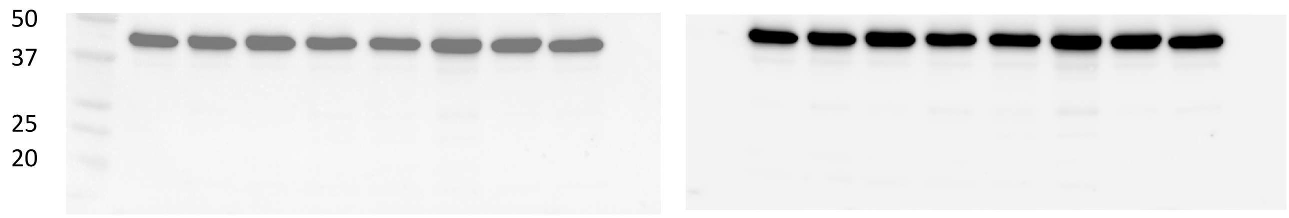

AC1G

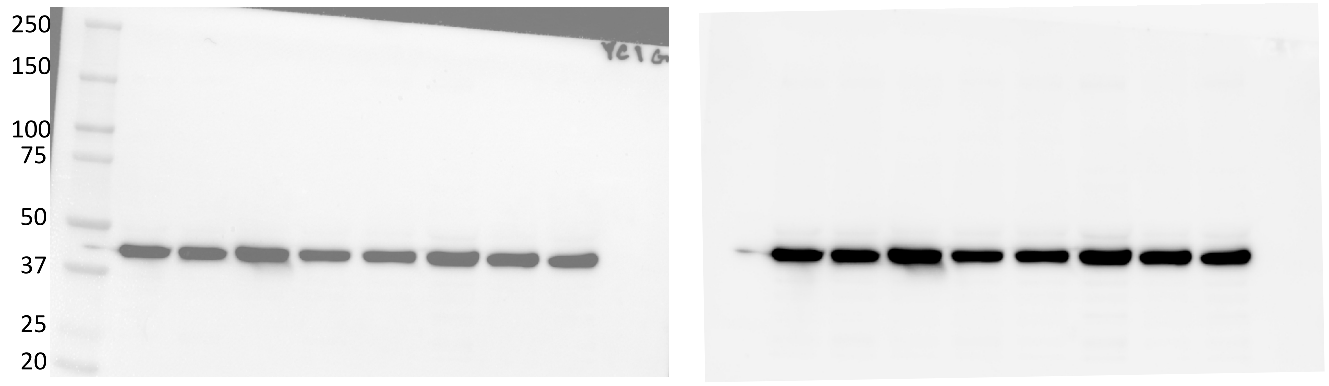

AC1H

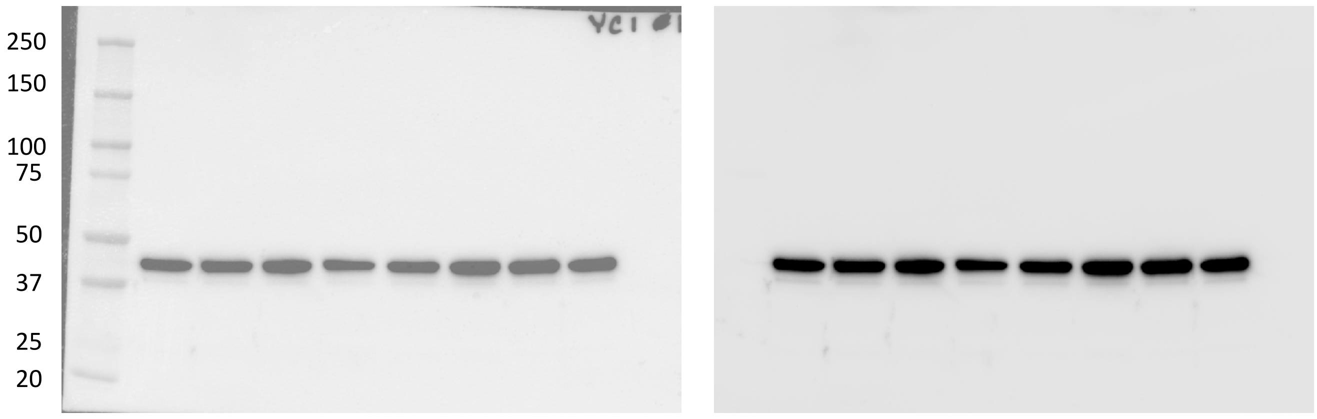

AC1 – R1

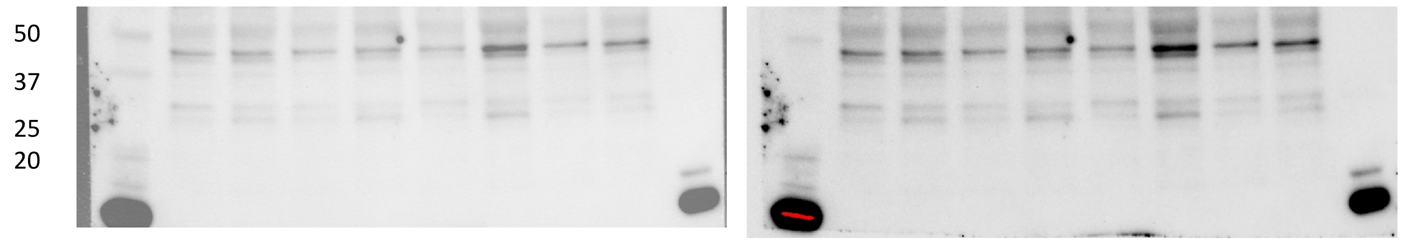

β-Actin – 42 kDa

Adolescent cortex (AC) – AC2

- 8 gels/blots
  - 4 normal run time
  - 4 extended run/separation
- Same loading
- Homogenate from rt. cortex

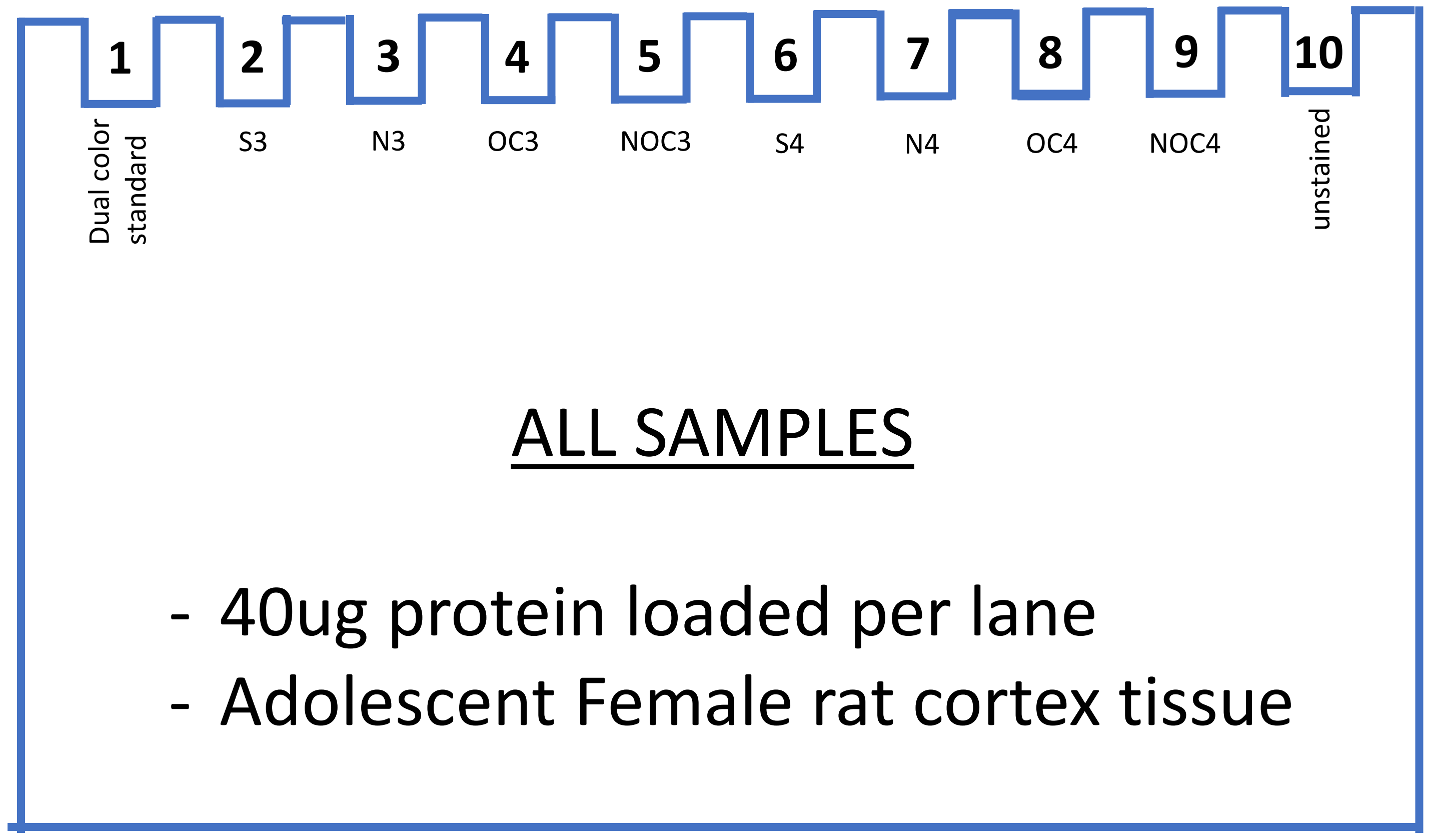

AC2

AC2F

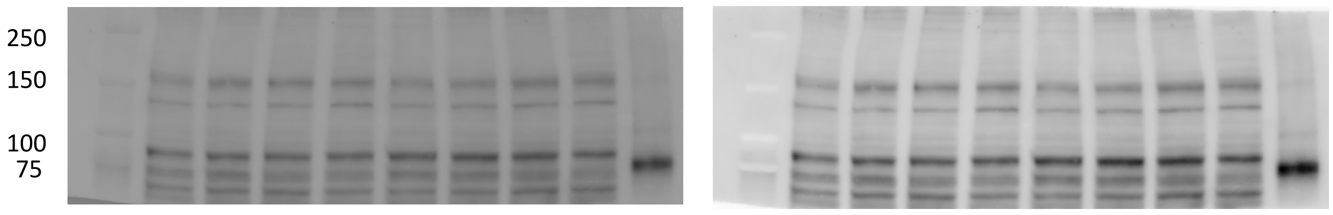

CPT 1 – 78 kDa

AC2E

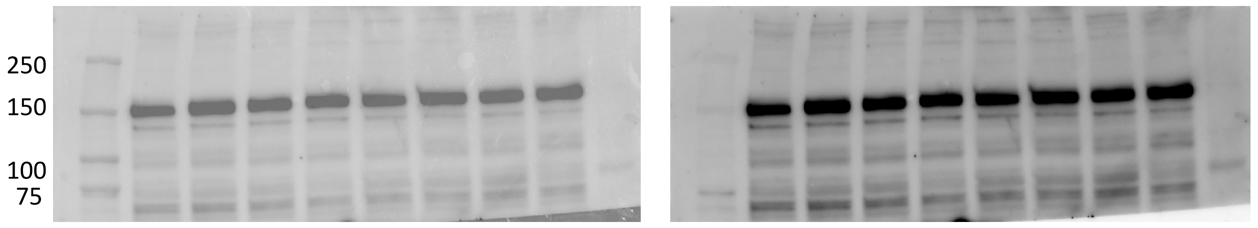

CPT 2 – 68 kDa

AC2A

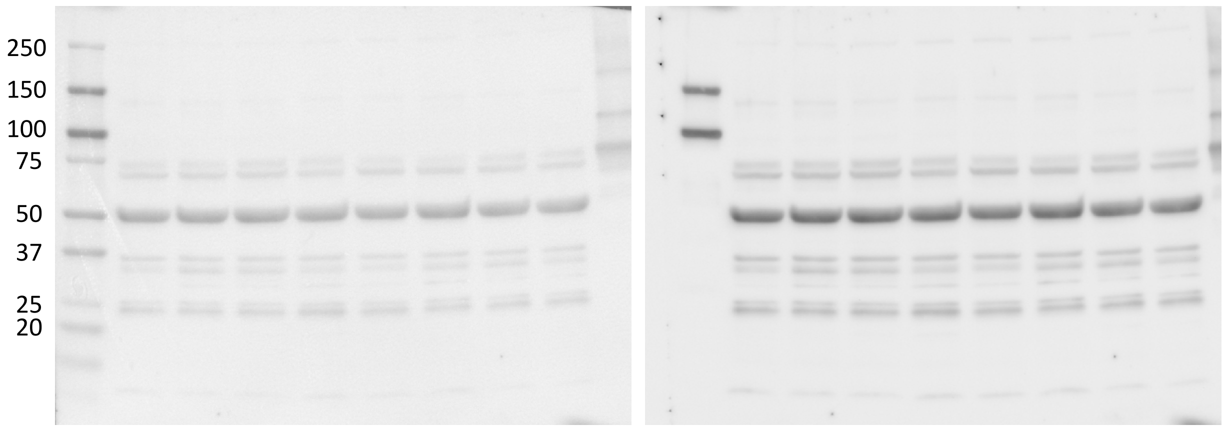

CKα – 52 kDa

AC2B

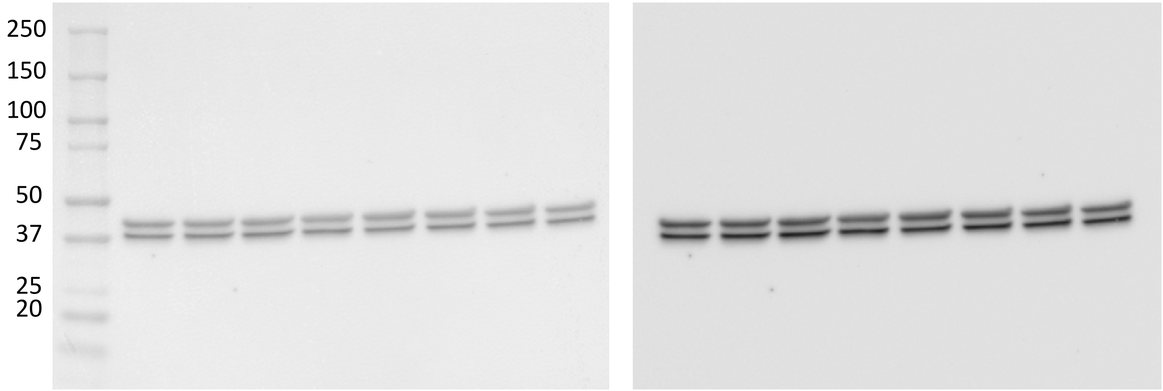

MAPK – 42,44 kDa

AC2H

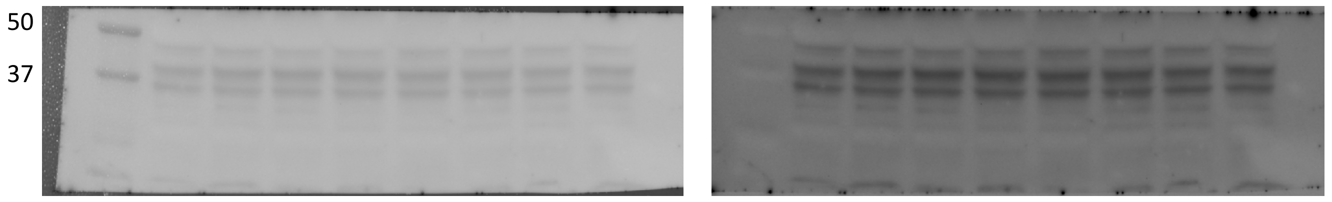

CCTβ (PCYT1B) – 42 kDa

AC2h – R2

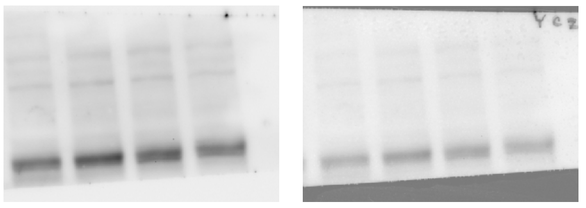

ACSL – 78 kDa

AC2 – R1

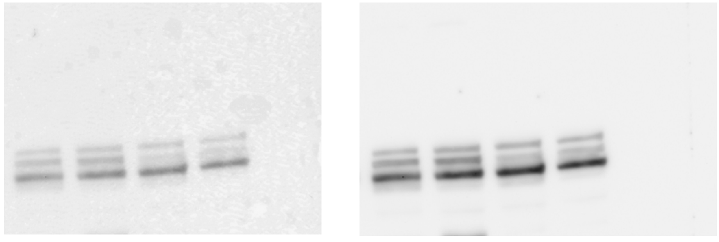

MLCD – 55 kDa

AC2 – R1

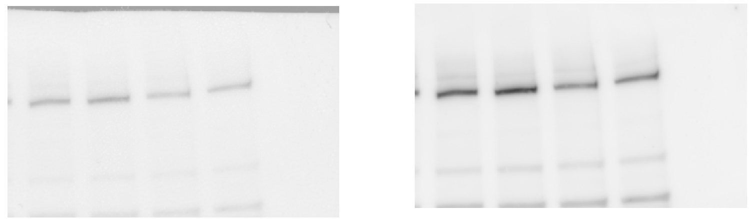

FASN – 273 kDa

AC2 – R1

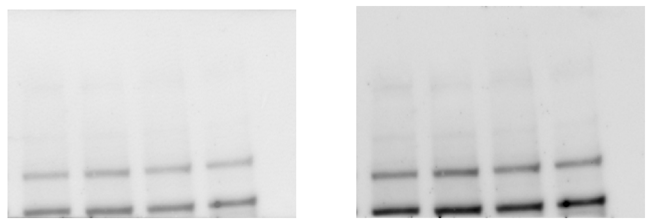

ACL – 125 kDa

AC2 – β-actin Blots

AC2A

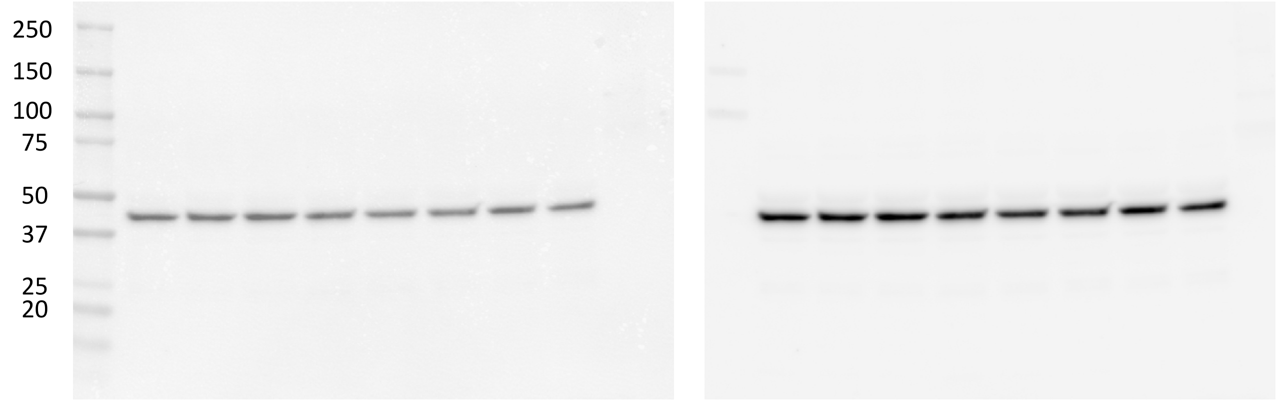

AC2B

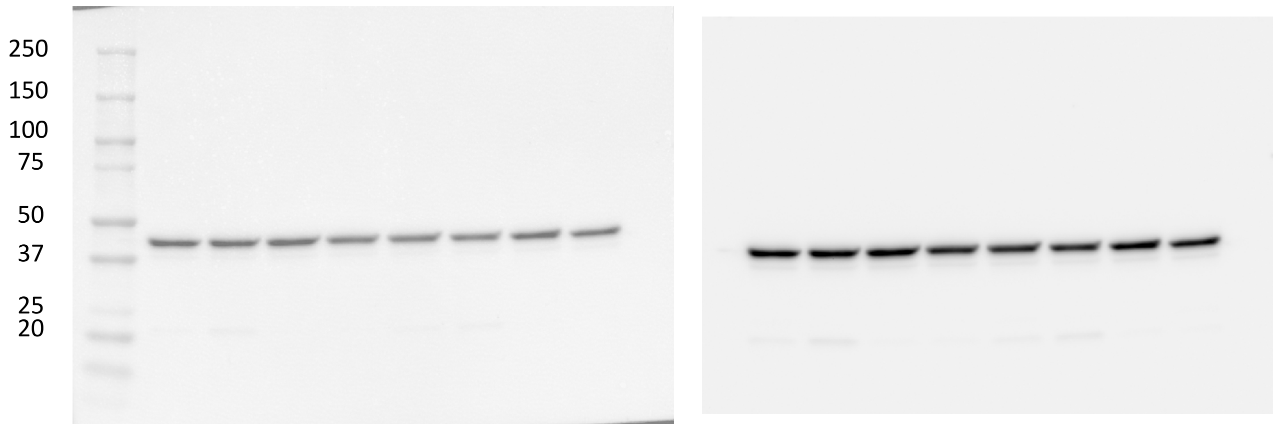

AC2E

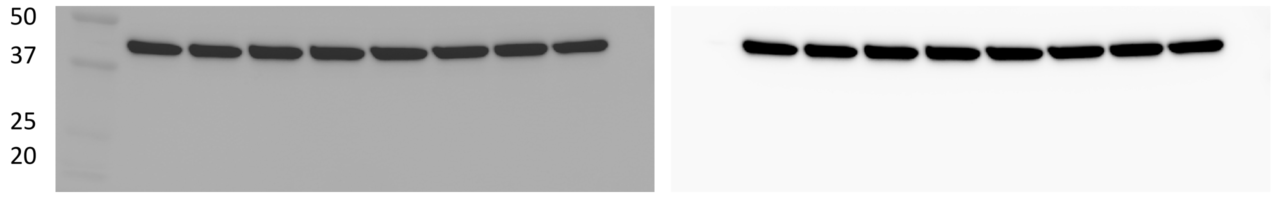

AC2F

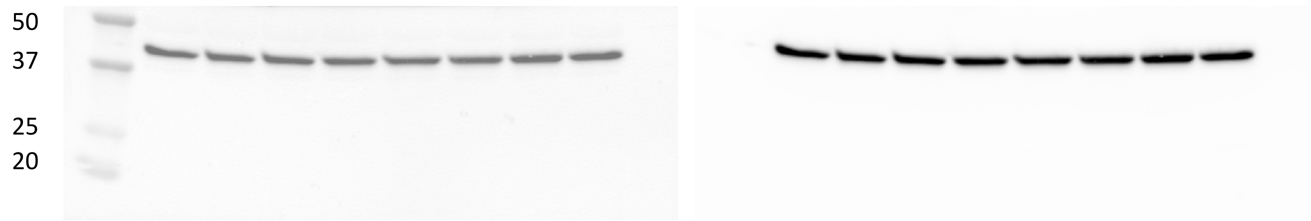

AC2H

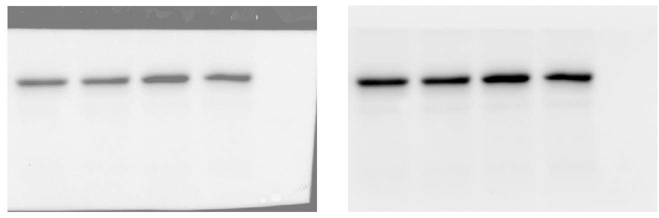

AC2 – R1

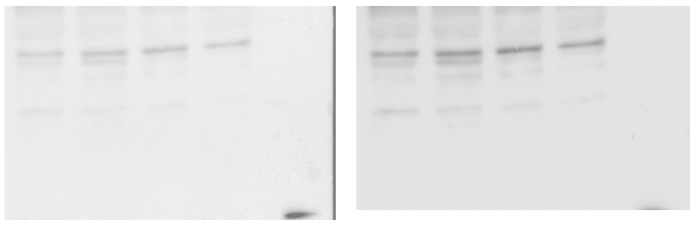

AC2 – R2

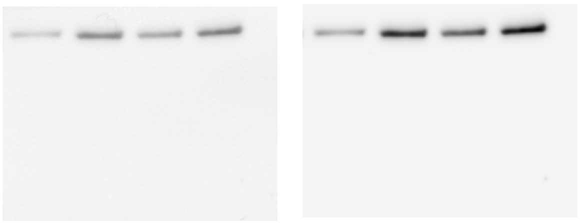

Adolescent cortex (AC) – AC3

- 8 gels/blots
  - 4 normal run time
  - 4 extended run/separation
- Same loading
- Homogenate from rt. cortex

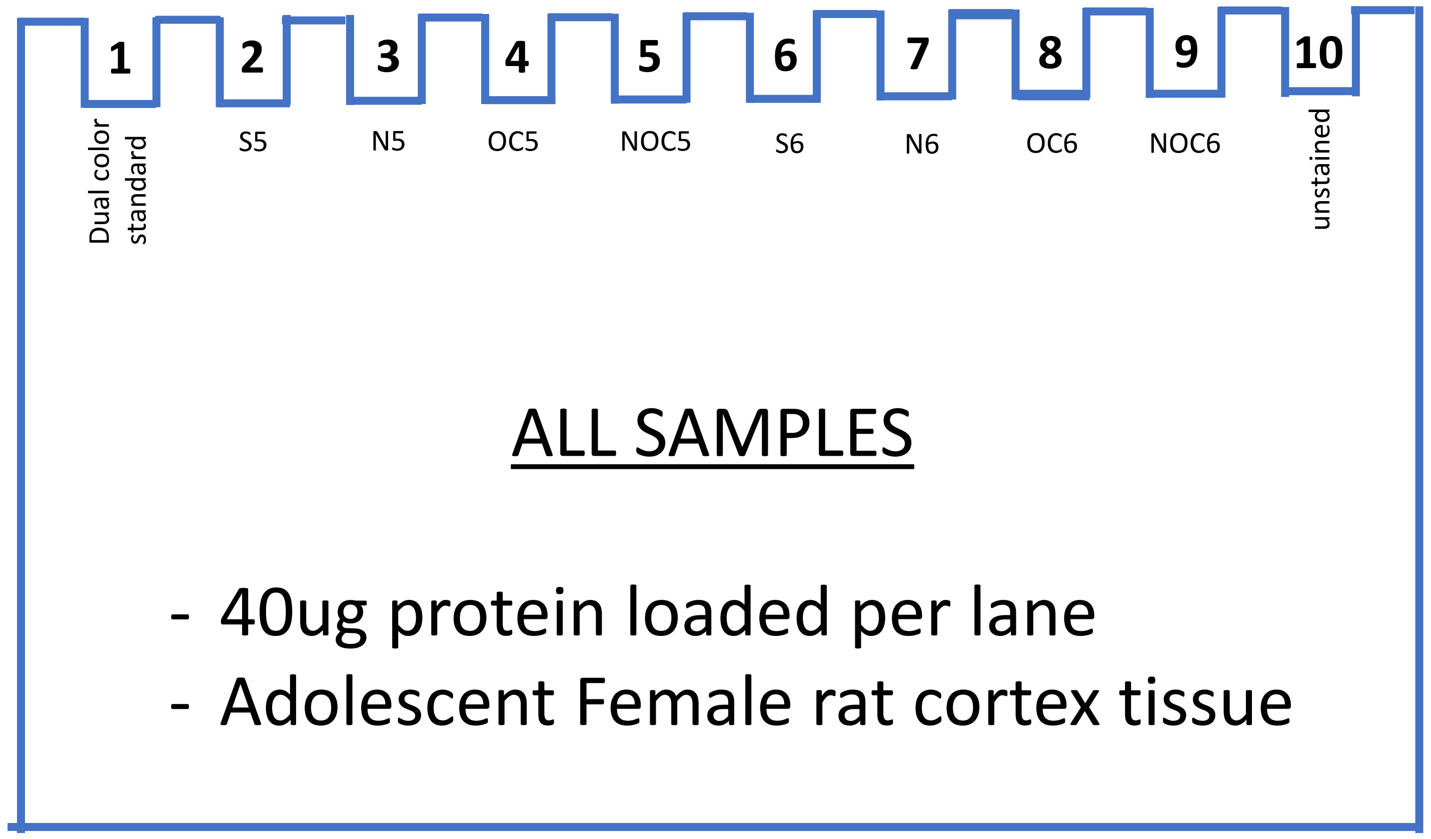

AC3

AC3G

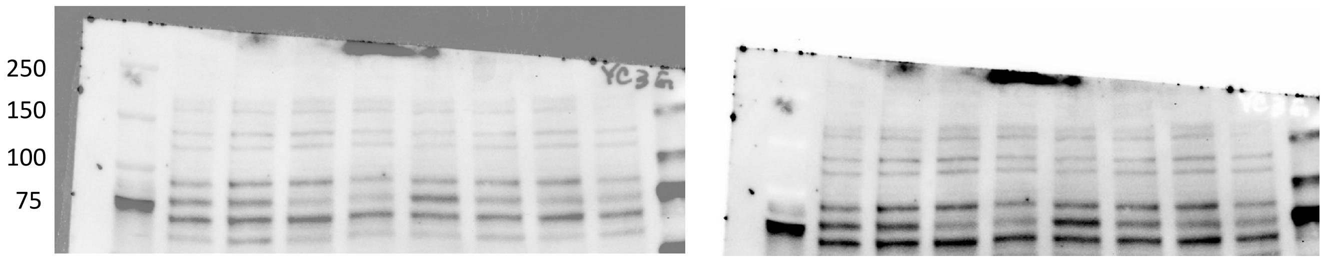

CPT 1 – 78 kDa

AC3N top

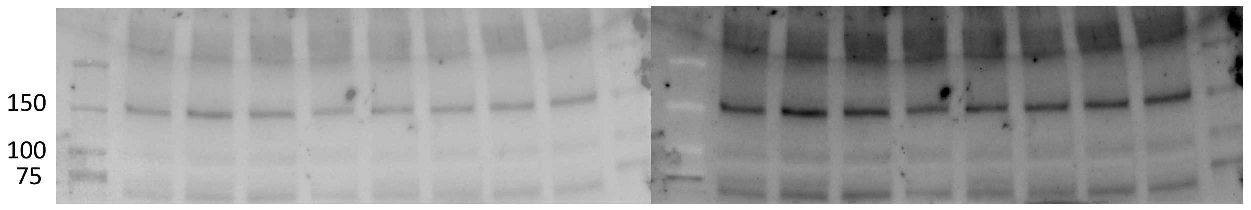

CPT 2 – 68 kDa

AC3E bottom

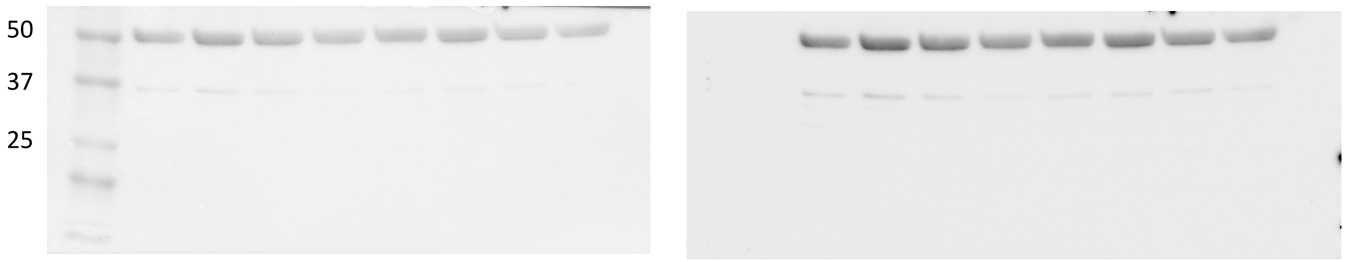

CKα – 52 kDa

AC3F bottom

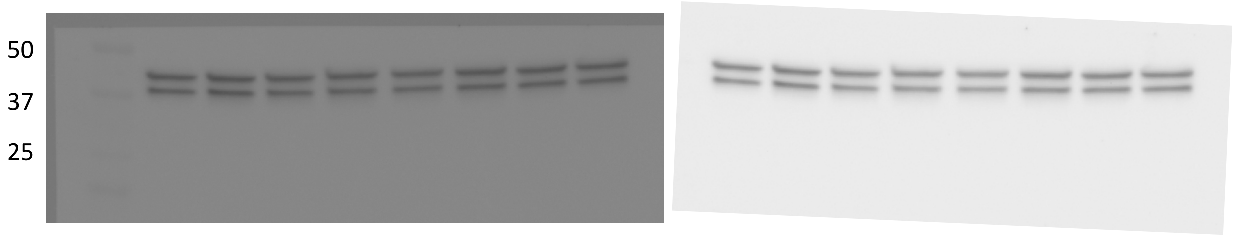

MAPK – 42,44 kDa

AC3Nb

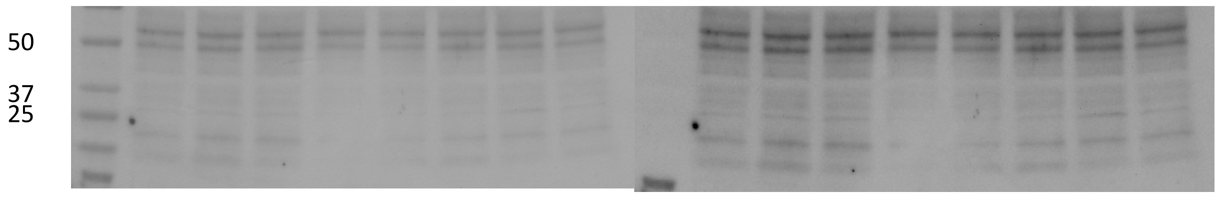

CCTβ (PCYT1B) – 42 kDa

AC3 – β-actin Blots

AC3E

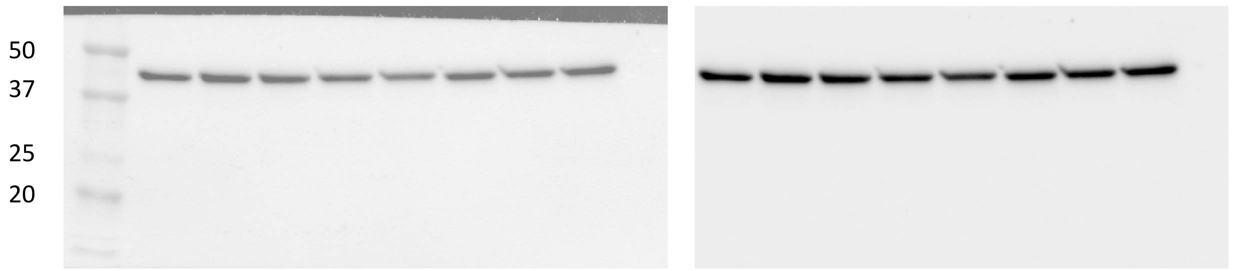

AC3F

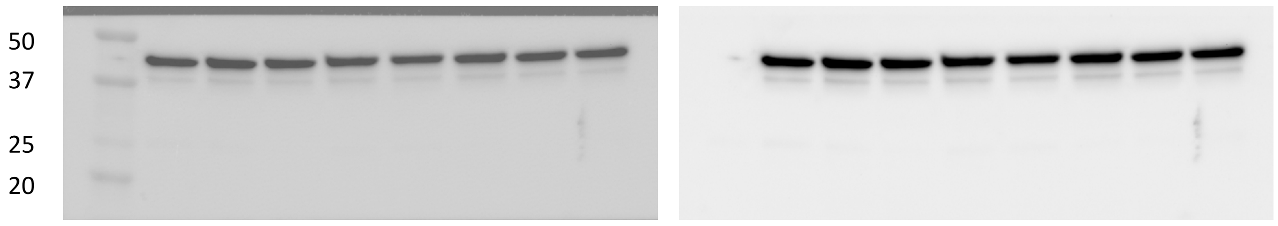

AC3D

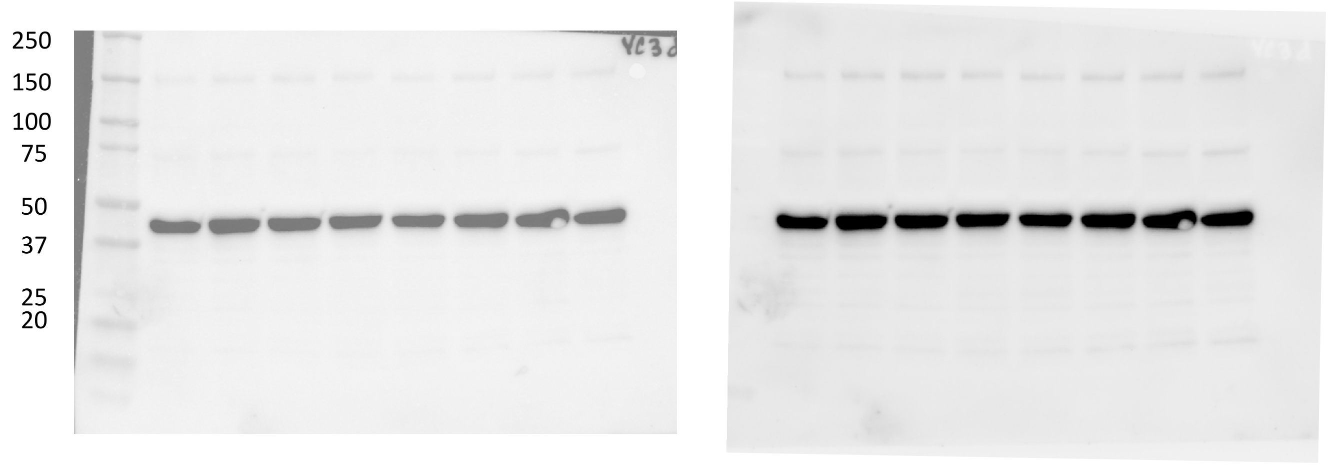

AC3G

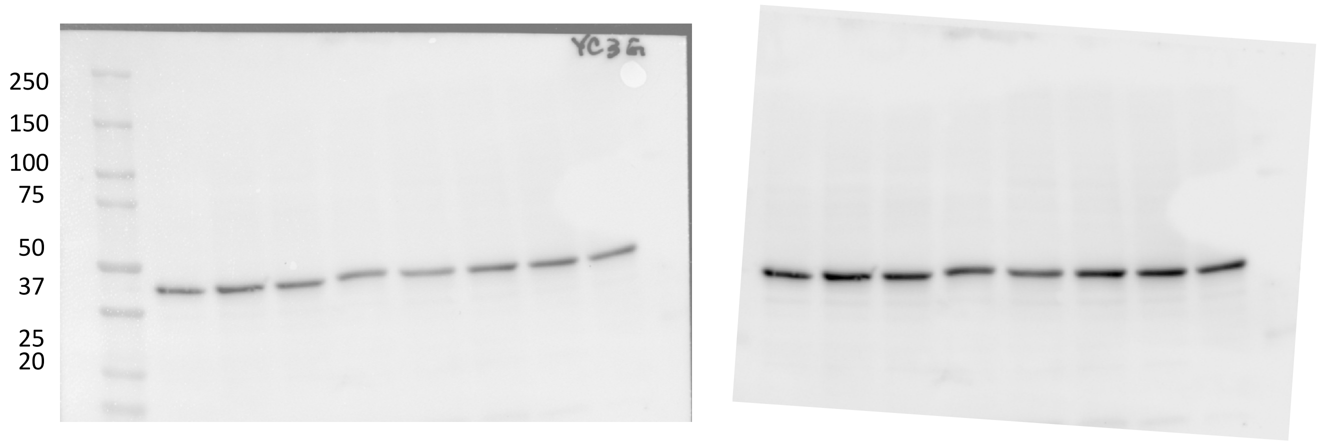

AC3N

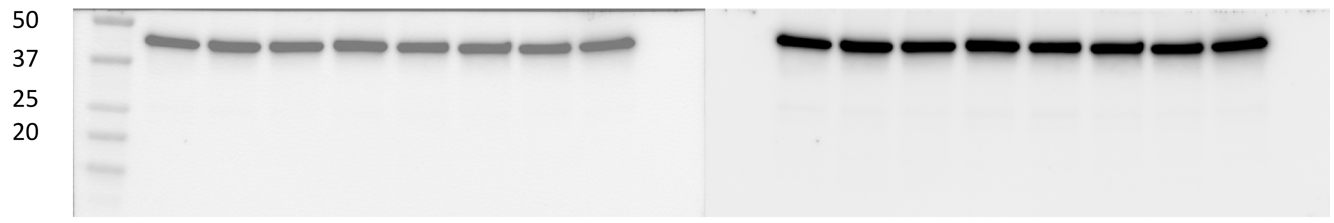

Adolescent cortex (AC) – AC4

- 8 gels/blots
    - 4 normal run time
    - 4 extended run/separation
- Same loading
- Homogenate from rt. cortex

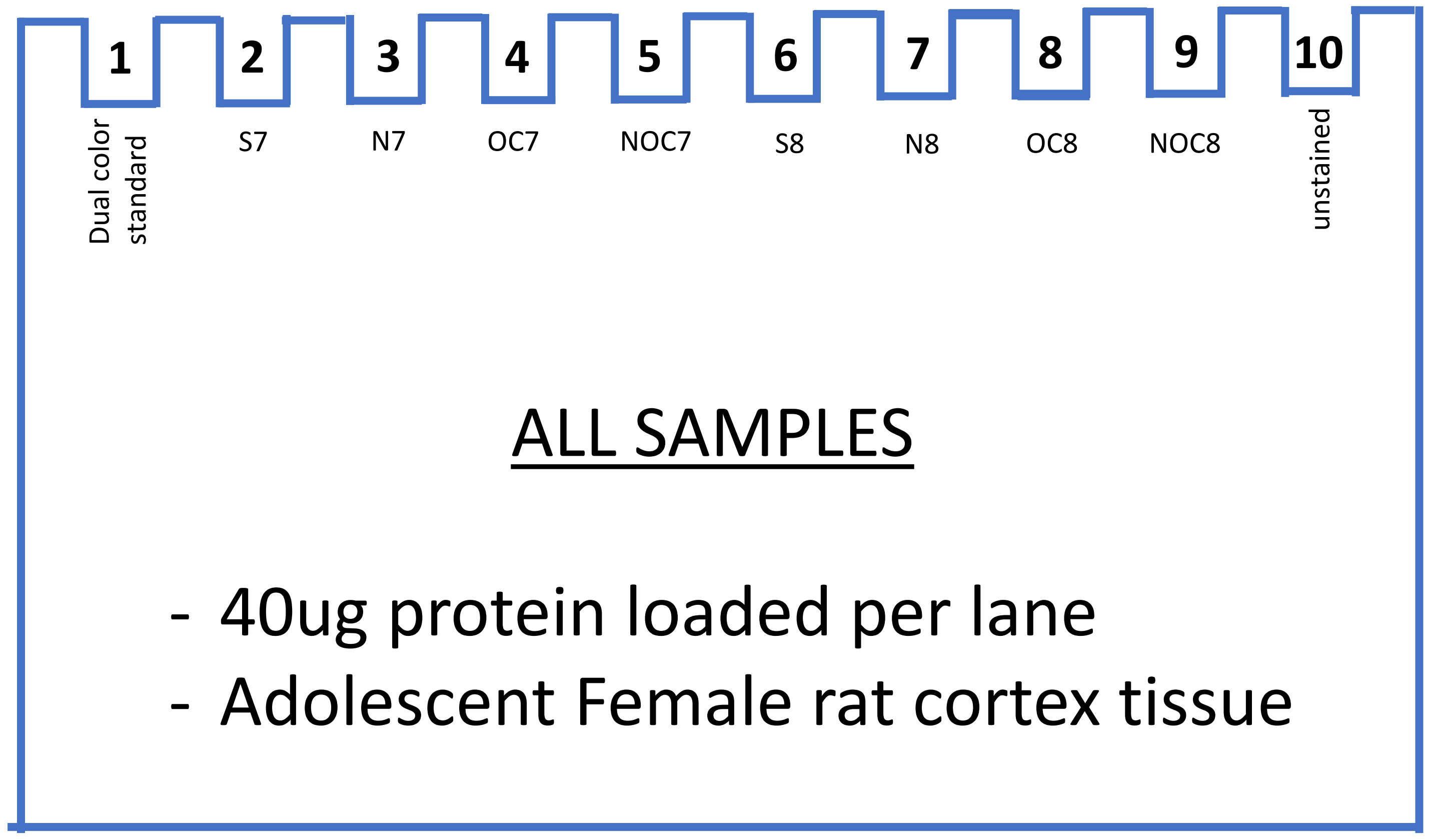

AC4

AC4E top

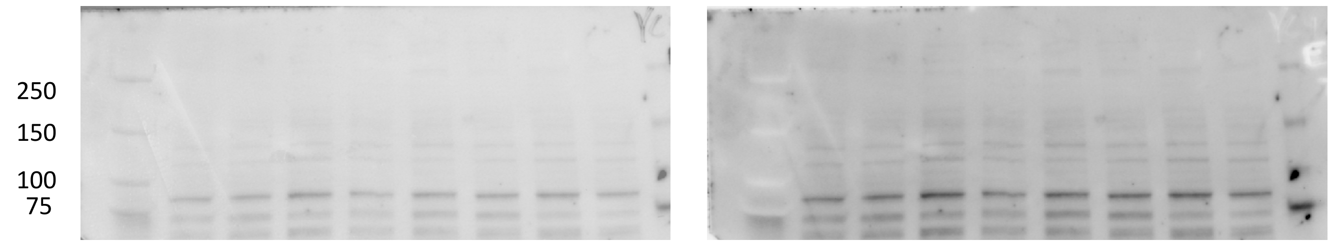

CPT 1 – 78 kDa

AC4E bottom

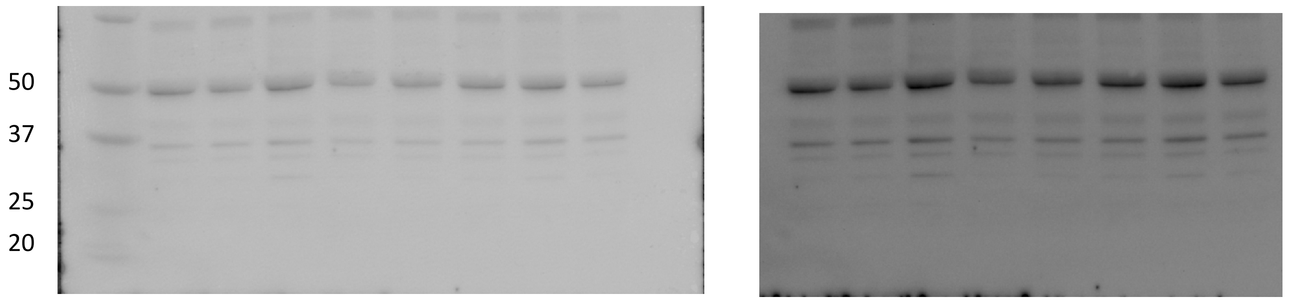

CKα – 52 kDa

AC4A bottom

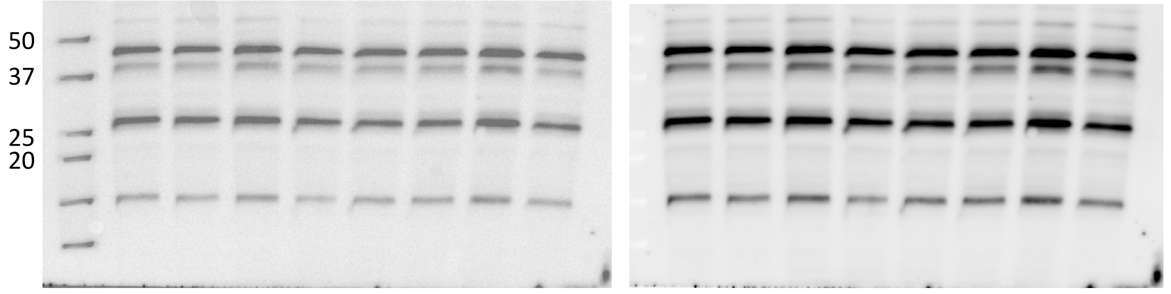

CCTβ (PCYT1B) – 42 kDa

AC4A top

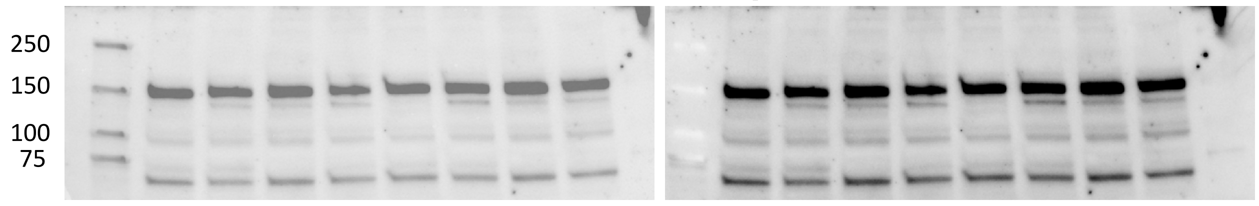

CPT 2 – 68 kDa

AC4F bottom

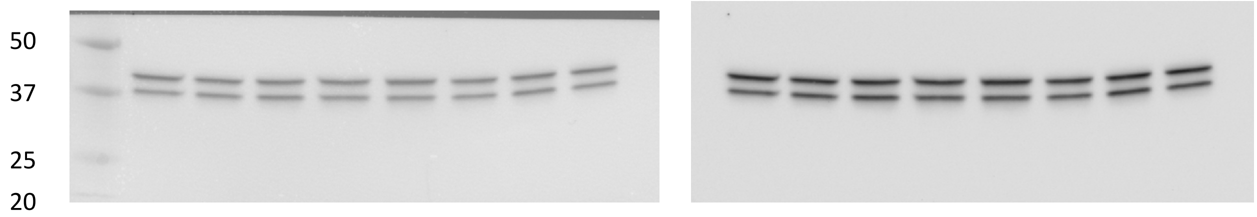

MAPK – 42,44 kDa

AC4L – R3

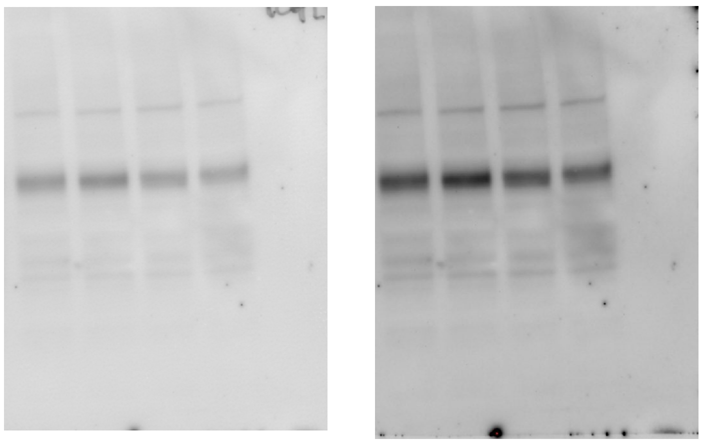

ACSL – 78 kDa

AC4bb – R1

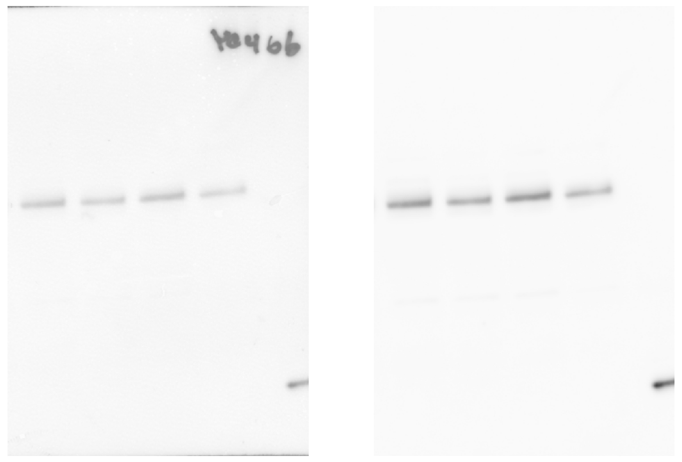

MLCD – 55 kDa

AC4bb – R1

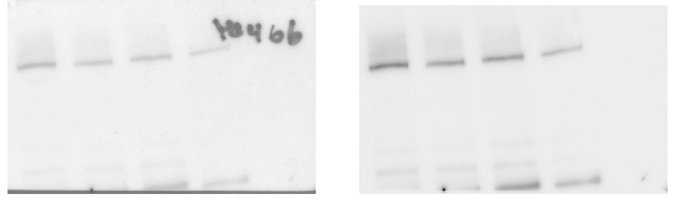

FASN – 273 kDa

YC4 – R2

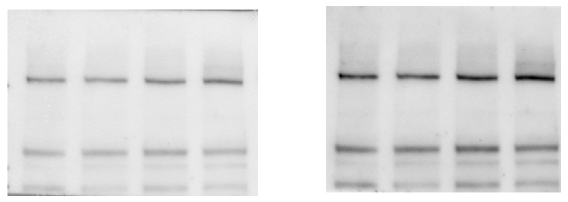

ACL – 125 kDa

AC4 – β-actin Blots

AC4A

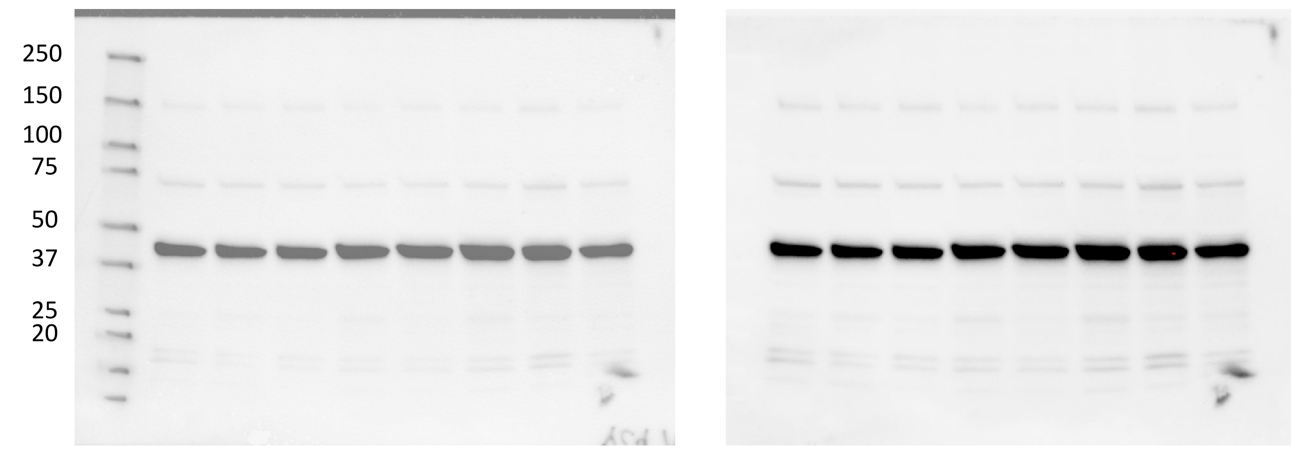

AC4B

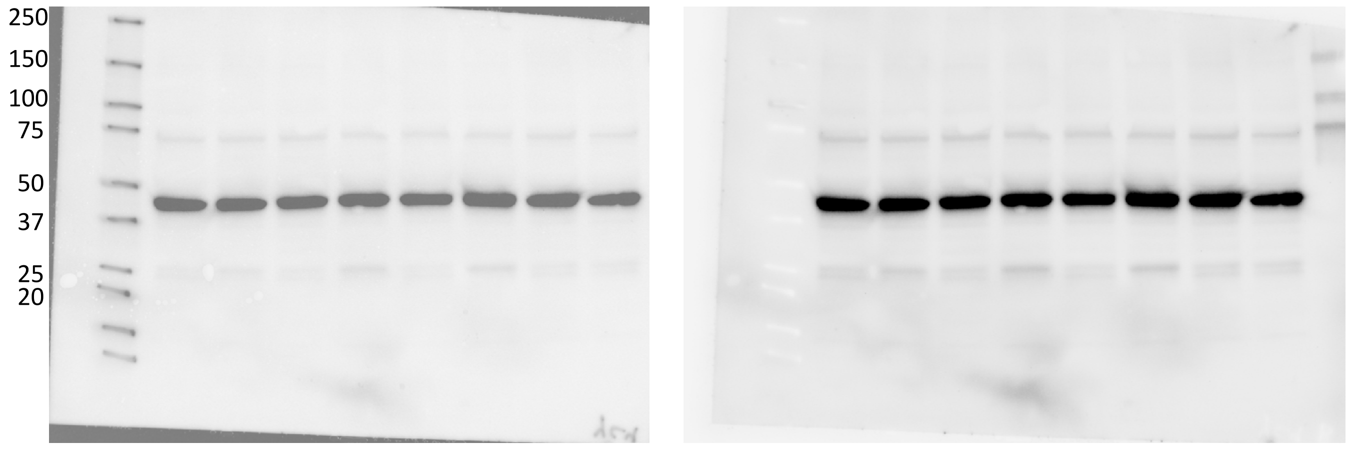

AC4G

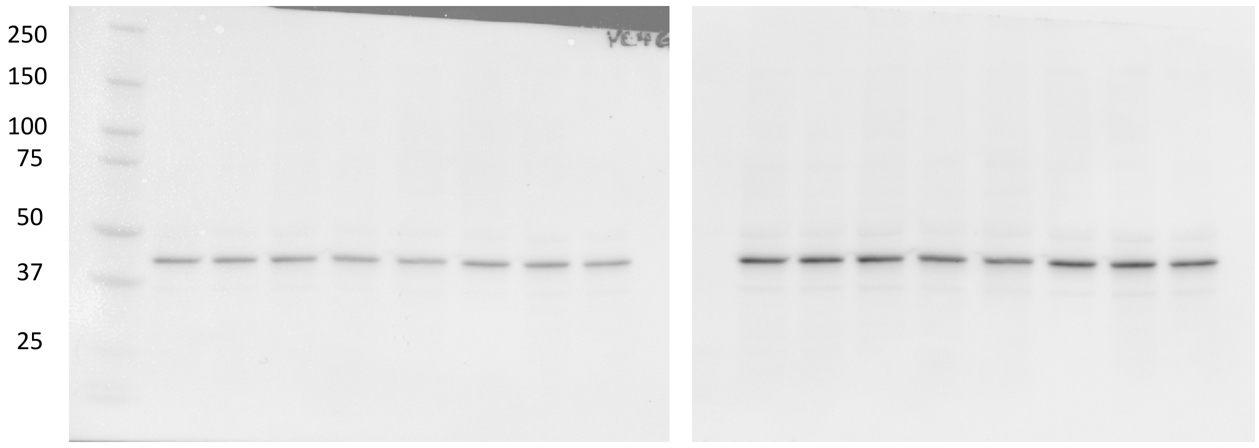

AC4E

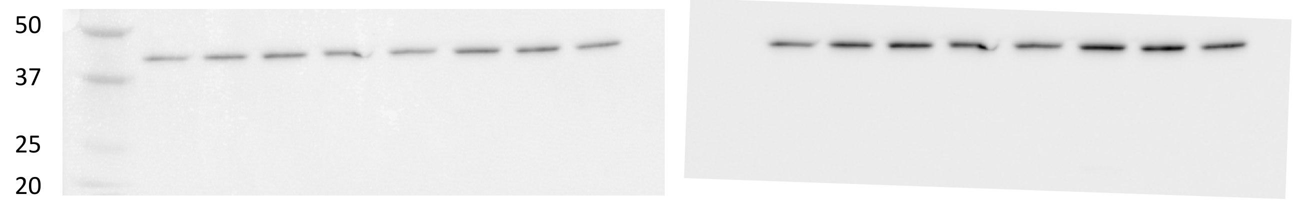

AC4F

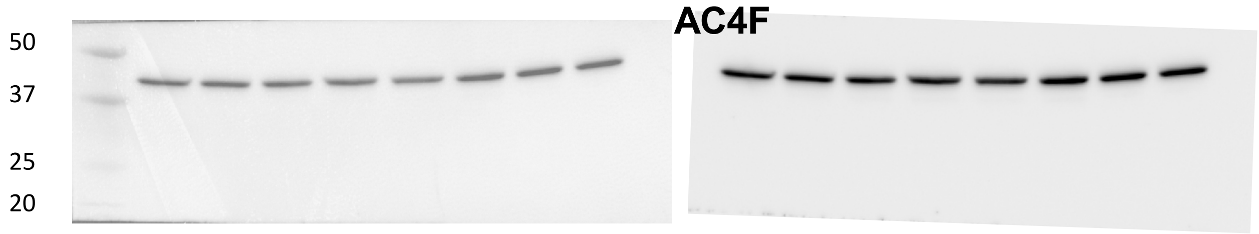

AC4L – R3

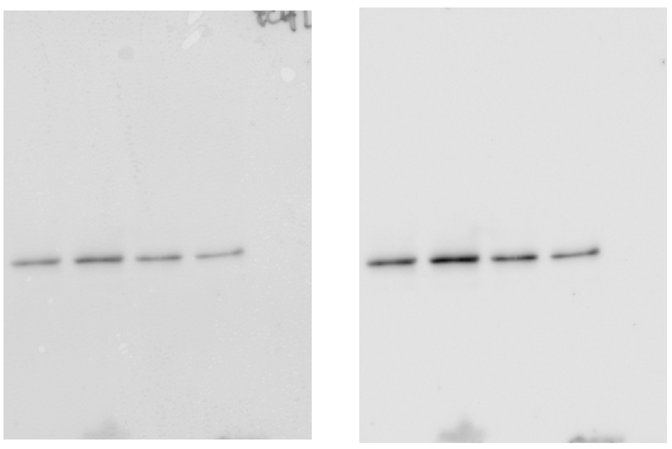

AC4bb – R1

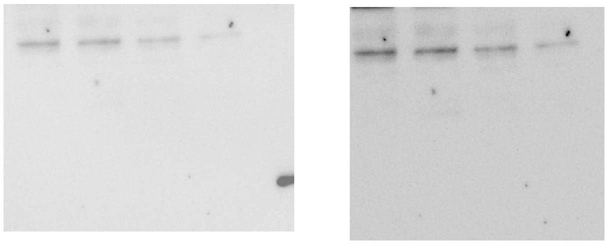

AC4 – R2

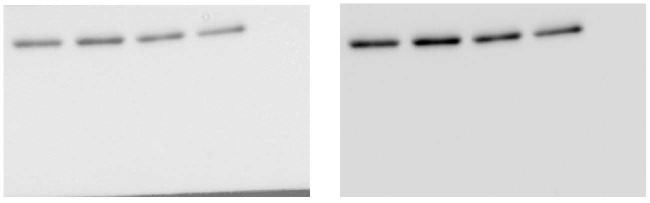

Supplement: Supplementary file 1 [file ijms-23-16075-s001.zip › Supplementary material/ijms-1962720-supplementary.pdf]
